# Data-driven burst shape analysis for functional phenotyping of neuronal cultures

**DOI:** 10.1016/j.isci.2026.117499

**Published:** 2026-09-21

**Authors:** Tim J. Schäfer, Emmanouil Giannakakis, Paul Schmidt-Barbo, Anna Levina, Oleg Vinogradov

**Affiliations:** 1 Department of Computer Science, University of Tübingen, 72076 Tübingen, Germany; 2 Max Planck Institute for Dynamics and Self-Organization, 37077 Göttingen, Germany; 3 Max Planck Institute for Biological Cybernetics, 72076 Tübingen, Germany; 4 Laboratory of Translational Immuno-Oncology, Department of Biomedicine, University and University Hospital Basel, 4031 Basel, Switzerland; 5 Collaborative Research Institute Intelligent Oncology (CRIION), 79110 Freiburg, Germany; 6 Department of Neurology and Epileptology, University of Tübingen, 72076 Tübingen, Germany

**Keywords:** burst shape, neural cultures, micro-electrode arrays, network dynamics, blocked inhibition, CACNA1A haploinsufficiency, rodent, human pluripotent stem cell

## Abstract

Cultures of neurons *in vitro* are instrumental for studying network dynamics under normal and pathological conditions. Mature networks typically exhibit network bursting activity, traditionally quantified by simplified features such as inter-burst intervals and burst durations. These features have advanced our understanding of development, disease phenotypes, and drug effects. However, they overlook the temporal structure within bursts, which is highly sensitive to network changes and can thus reveal additional physiological or pathological effects. Here, we developed a comprehensive framework to quantify burst shapes, the time-course of network firing during bursts. On four datasets, including rodent- and human pluripotent stem cell-derived cultures, we show that burst shapes contain rich information about the underlying network dynamics. We quantify this information by classifying conditions (genetic disorders, pharmacological agents) using traditional and burst-shape features, and find that including shape features significantly improves accuracy. We provide an open pipeline for burst shape characterization, introducing burst shape as a complementary functional phenotyping feature that expands the analytical toolkit for disease modeling and drug screening.

## Highlights

• Improved description of population bursting activity in primary/derived neuronal cultures
• Data-driven framework for quantifying burst temporal profiles (burst shapes)
• Combining burst shape and traditional features consistently improves phenotype separability

## Graphical abstract

**Figure:**
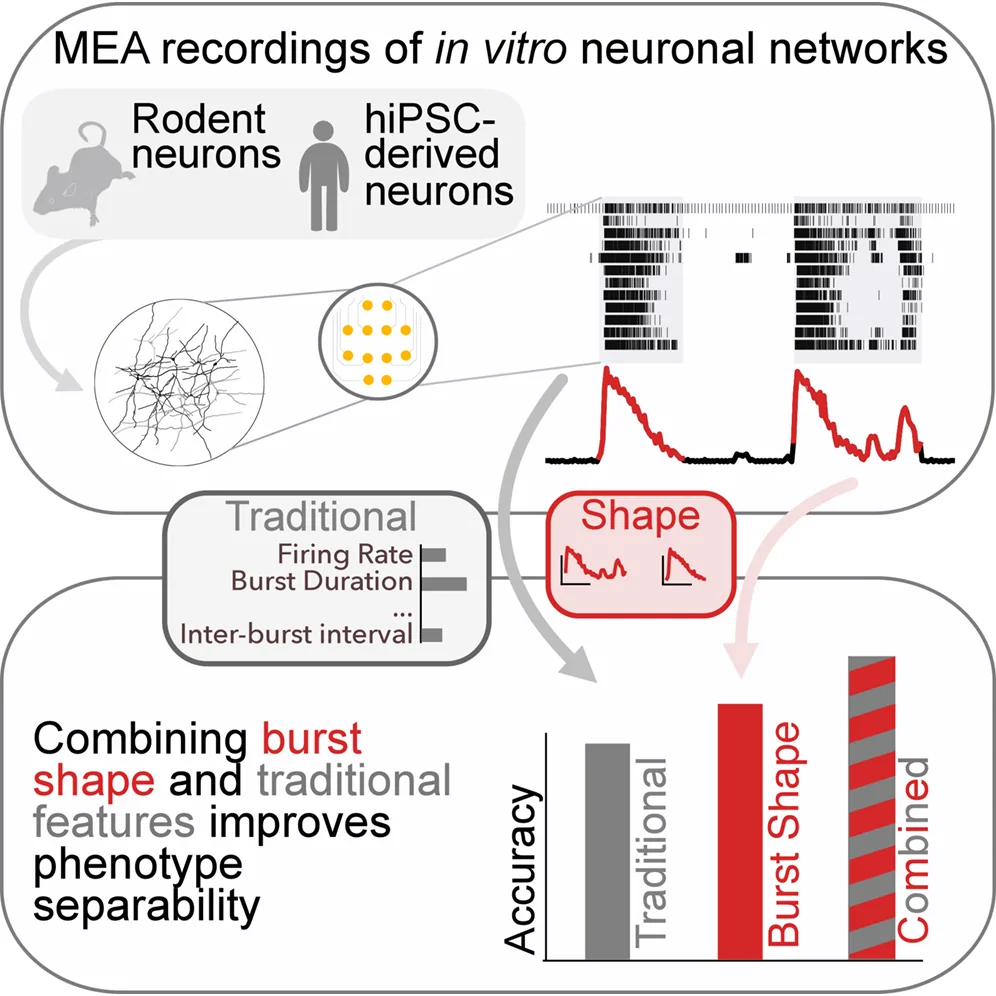

## Teaser

Neuroscience; Machine learning

## Introduction

Synchronized network bursts are a fundamental activity mode of developing neural networks *in vivo* and *in vitro*, arising from the interplay of excitability, connectivity, and dynamic neuron characteristics.^1^^,^^2^^,^^3^^,^^4^^,^^5^^,^^6^ Neural cell cultures often develop such bursting activity, typically characterized by synchronous firing lasting from tens of milliseconds to a few seconds, followed by long, irregular quiescent intervals. Such activity patterns are observed in cortical, hippocampal, striatal, and spinal cord cultures *in vitro* as well as cultures of human induced pluripotent stem cell (hiPSC)-derived neurons.^4^^,^^7^^,^^8^^,^^9^^,^^10^ Deviation of bursting dynamics statistics from healthy controls *in vitro* is a strong indicator of neuro-developmental disorders.^11^^,^^12^^,^^13^^,^^14^

Traditionally, population activity in neuronal cultures is characterized by summary statistics of bursting episodes, such as duration, inter-burst interval, and average firing rate.^6^^,^^8^^,^^15^^,^^16^ With the ever-growing availability of tools to monitor network activity,^17^^,^^18^^,^^19^ these measures have become a common instrument to assess the effects of disease-associated gene variants, as well as chronic and acute perturbations on individual neurons and networks.^9^^,^^10^^,^^11^^,^^13^^,^^20^^,^^21^^,^^22^^,^^23^^,^^24^^,^^25^

However, the burst shape, the detailed time course of firing rate within the burst, has received comparatively little attention, even though theory, simulations, and experiments all point to a close link between network properties and intra-burst dynamics.^7^^,^^12^^,^^26^^,^^27^^,^^28^ Previous works attempted parametric descriptions of burst shapes tailored to individual experiments, including features such as rise, peak, and decay time.^10^^,^^15^^,^^25^^,^^29^ While informative, these simple metrics are limited in two key aspects. First, they oversimplify complex activity patterns, such as fluctuating firing rates, rebound firing, and oscillations. Second, summarizing burst shapes into a few numbers per recording conceals the variability of burst shapes within each culture, potentially obscuring biologically meaningful heterogeneity.

To overcome these limitations, we introduce a data-driven framework for normalizing and comparing burst shapes in a standardized way. Applied to four published datasets of rodent- and hiPSC-derived neural cultures,^10^^,^^15^^,^^25^^,^^30^ this framework shows that burst shape information significantly improves the classification of experimental conditions, revealing a close link between intra-burst dynamics and underlying network properties. Our approach enables visualization of burst shapes and extraction of compact, interpretable descriptors that approach the performance of the full data-driven representation. Additionally, we find that genetically similar cultures exhibit similar burst shape patterns, demonstrating that burst shape carries complementary, biologically relevant information about network phenotype.

## Results

We aim to maximize the information extracted from multi-electrode array (MEA) recordings obtained under diverse experimental conditions, including pharmacological manipulations and disease-related mutations (Figure 1A). Traditionally, population activity is described by a set of simple statistical features such as burst duration, inter-burst interval, and firing rates (Figure 1B). Because these measures overlook the temporal structure within bursts, we propose to augment them with burst shape features. In the following, we describe how burst shapes can be represented and compared, and how these representations can be incorporated into statistical models trained to infer experimental conditions. Within this framework, the improvement in classification accuracy quantifies the additional information carried by burst shapes (Figure 1C).

**Figure 1:**
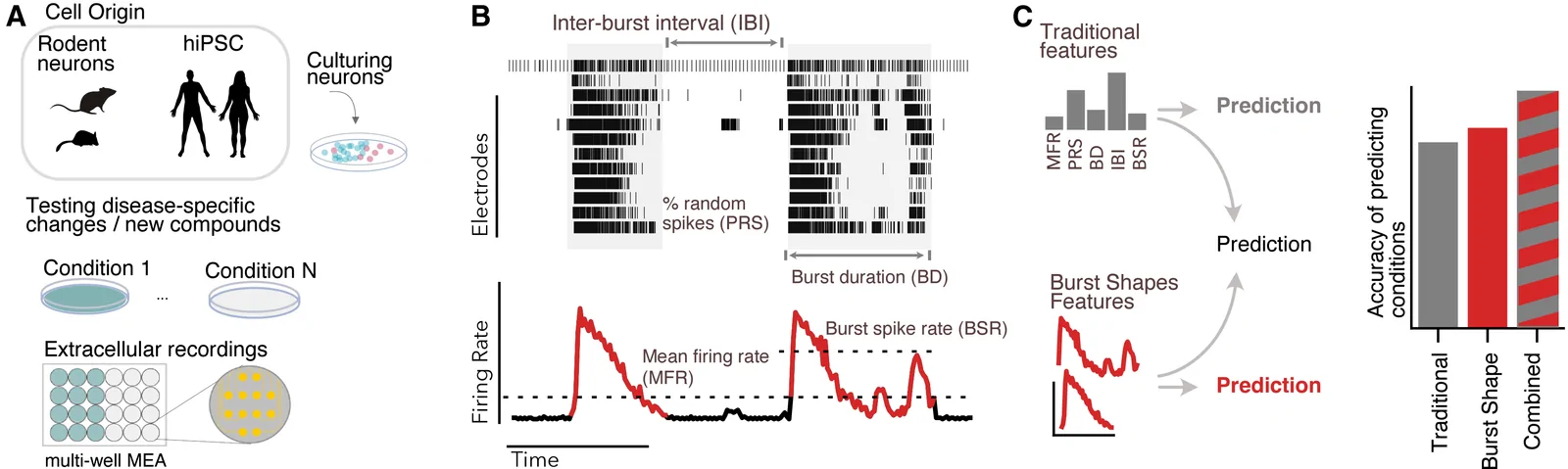
Schematic of evaluating burst shapes as complementary to traditional features for network phenotyping (A) Neural cell cultures, including rodent- and hiPSC-derived cells, plated and recorded with multi-electrode arrays (MEAs). (B) Traditional descriptors of bursting activity (inter-burst intervals, burst duration, percentage of random spikes, firing rate overall, and during bursts) are complemented by burst shape features. (C) The contribution of traditional and burst shape features is assessed using models inferring the experimental condition from the different feature subsets.

### Framework for data-driven quantification of burst shapes

We analyzed four datasets of MEA-recorded network activity from neural cultures under various experimental conditions (dataset short names in brackets, see STAR Methods for details): mouse cortical neurons with inhibition blocked by the GABA_A_ receptor antagonist bicuculline (blocked inhibition),^30^ hiPSC-derived neurons with *CACNA1A* haploinsufficiency (*CACNA1A*^+/−^),^25^ hiPSC-derived neurons from Kleefstra syndrome patients and controls (Kleefstra),^10^ and rat cortical neurons in plating batches (developing rat).^15^ We detected 31,949 network bursts in 970 recordings and 604 h of activity (Table S1). To ensure reliable burst detection at this scale, we developed an interactive tool for detailed review and parameter tuning (Figure S1, detection parameters summarized in Table S2, alternative burst detection in Table S4).

To compare burst shapes across a wide range of durations (tens of milliseconds to several seconds) and network firing rates, we standardized all burst shapes to unit duration and unit integral (Figure 2A). Averaging these standardized shapes provides an interpretable profile of group differences, enabling the design of manual burst shape features (Figure 2B). We capture the similarity between individual bursts by constructing the K-nearest neighbor (KNN) graph, linking each burst shape to its *K* most similar burst shapes, measured with the Wasserstein distance (also called Earth Mover’s distance) (Figure 2C, see STAR Methods). From this graph, recording-level predictions were obtained by aggregating the neighbors of individual bursts (Figure 2D). We then applied spectral embedding to the KNN graph, preserving its structure while projecting burst shapes into a low-dimensional space (Figure 2E), which can be explored interactively (Figure S2). The low-dimensional embeddings across datasets have correlations with conventional burst features, such as burst skewness, peak time, and burst width. However, the strength and sign of these correlations are variable across datasets (Figures S14 and S15). Averaging the two-dimensional representation of all bursts in the recording yielded data-driven shape summary features (Figure 2E). Finally, we combined these shape features with the traditional features in an XGBoost classification (Figure 2F), comparing the performance between classifiers. This representation of burst shapes provides the basis for evaluating their contribution to classifying experimental conditions, as detailed in the following section.

**Figure 2:**
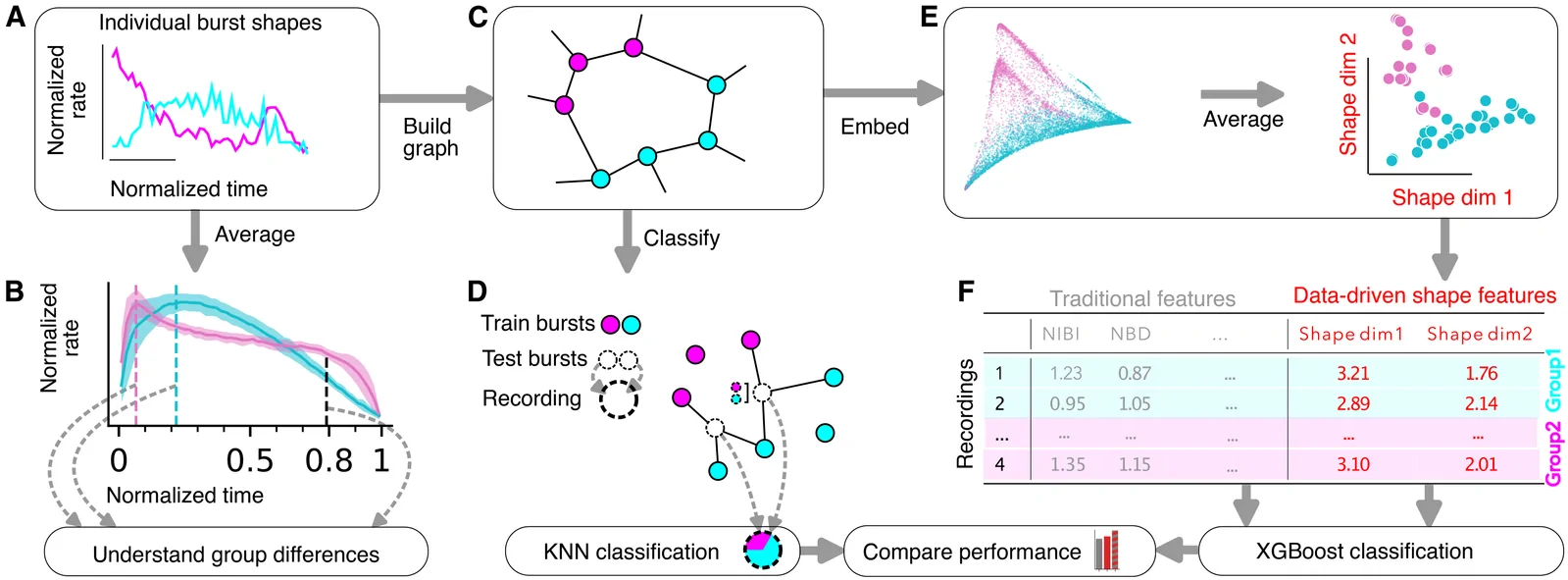
Framework for data-driven quantification of burst shapes (A) Standardized burst shapes are computed by normalizing duration and size. (B) Understanding the group differences by averaging across recordings. (C) KNN graph connects each node (burst shape) to the *K* most similar nodes. (D) Classifying recordings based on individual burst shapes’ neighbors aggregated to the recording level. (E) Spectral embedding of individual burst shapes is aggregated (averaged) to the recording level, where the shape dimensions define the shape features. (F) Traditional and shape summary features are combined to predict recording identities using XGBoost.

### Augmenting traditional features with burst shape features improves classification performance across datasets

To test which features contain meaningful information about culture phenotypes, we trained four classifiers: KNN classification using individual burst shapes (Figure 2D) and XGBoost classification using three feature sets: traditional, burst shape, and combined (Figure 2F), where all models performed well above chance level (Figure 3A). We carefully compared the cross-validated model performances with the frequentist approach (see STAR Methods)^31^^,^^32^ and report Bayesian comparison in Table S3 and Figure S6. Generally, including burst shape information improved classifier performance, providing a more discriminative description of culture phenotypes within the studied datasets.

**Figure 3:**
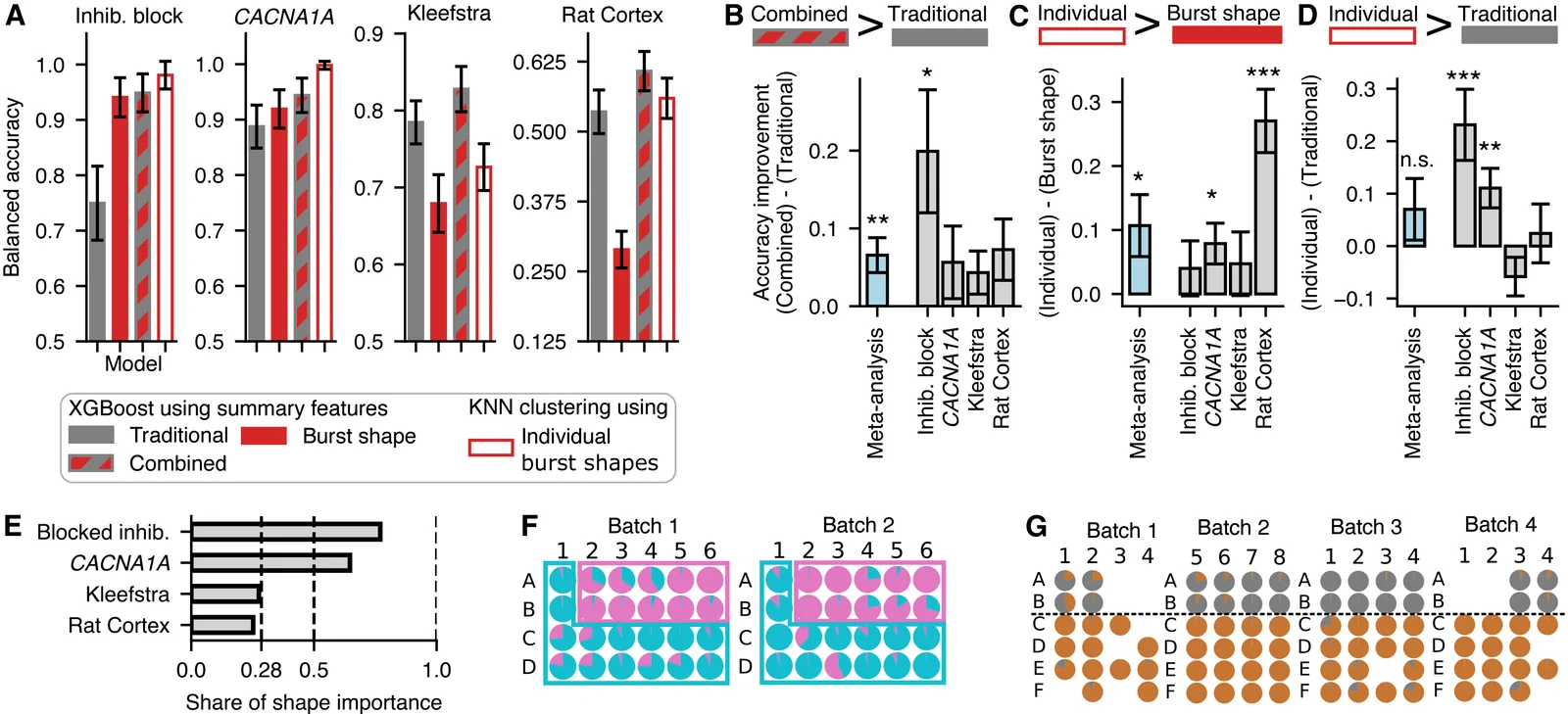
Augmenting traditional features with burst shape features improves classification performance across datasets (A) Mean classification accuracies for four models: KNN clustering based on individual burst shapes, and XGBoost models based on three summary feature sets (traditional, shape-2D, and combined). Data are represented as mean ± standard error of the mean (*n* = 100 shuffle splits, 80–20 ratio); *y* axis minima represent chance levels. (B–D) Difference in accuracies between models trained on datasets indicated in axis label. Combined feature set outperforms the traditional set (B), individual burst features outperform shape summary features (C), and in some cases outperform traditional features (D). Data are represented as mean ± standard error of the mean (*n* = 100 shuffle splits, 80–20 ratio). Stars indicate significance with Wald test (∗*p* < 0.05, ∗∗*p* < 0.01, ∗∗∗*p* < 0.001); meta-analysis (hypothesis stated on top of each image) used a random-effects model. (E) Shapley value analysis of the seven combined features shows that the two shape features substantially contribute to prediction. (F) KNN classification predictions (leave-one-out) for the blocked inhibition dataset in the layout with 4 × 6 wells per batch. Pie-chart shares represent the weighted neighbors of the recording; solid borders indicate the true class. (G) KNN classification predictions for the *CACNA1A* dataset. The dashed line separates the true labels (top: control, bottom: *CACNA1A*^+/−^).

The combined feature set (burst shape and traditional descriptors) is more informative than the traditional features alone, as indicated by the significantly improved classifier accuracy (meta-analysis, *p* = 3.4 × 10^−3^, Figure 3B; strongest for the Wasserstein metric, see STAR Methods; Figure S10). To identify which of the seven combined-set features contributed most to this improvement, we computed Shapley values^33^ (Figure S7). Burst-shape features contributed strongly, accounting for more than 50% of the importance in the blocked-inhibition and *CACNA1A* datasets (Figure 3E), and about 30% in the two other datasets.

The KNN graph-based classifier directly leverages the similarity between individual bursts from different recordings. In some pharmacological and disease datasets, this similarity provided a good characterization of the culture phenotype, achieving near-perfect accuracy in blocked inhibition (98%) and *CACNA1A* data (100%), and showing high model confidence (Figures 3F and 3G, other datasets in Figures S4 and S5). Compared with the representation based on summary burst-shape features, the individual burst-shape similarities contained significantly more information about the experimental condition (Figure 3C, *p* = 2.3 × 10^−2^).

Taken together, the two approaches address distinct analytical objectives. The KNN-based classifier is most suitable for label transfer and classification between known conditions within the same dataset, as it directly leverages similarities between individual bursts. In contrast, the burst-shape embedding provides a flexible representation that can be integrated with traditional features, enabling richer characterizations of culture phenotypes and supporting exploratory and comparative analyses across datasets.

### Averaged normalized burst shapes reveal altered onset and offset dynamics

While data-driven shape features from spectral embedding are compact and perform well in classification, their abstract dimensions are not readily interpretable, providing only limited guidance for studying the underlying mechanisms. To address this, we designed features from averaged burst shapes that capture biologically meaningful aspects of onset and termination dynamics while retaining predictive performance close to that of data-driven features.

Burst shape differences between groups are visible from representative examples of raw (not normalized) network bursts (Figure 4A). However, raw burst shape averages (Figures 4B and S3A–S3C) are dominated by large, long bursts, obscuring shape features. In contrast, the averages of normalized burst shapes have very tight group means (Figure 4C, shading indicates ±3⋅standard error of the mean). This highlights the importance of normalizing burst shapes to unit duration and unit integral as a key step in the analysis. The altered onset and offset dynamics are the most prominent shape differences between conditions, which we capture with two manually designed shape features (dashed lines in Figure 4C).

**Figure 4:**
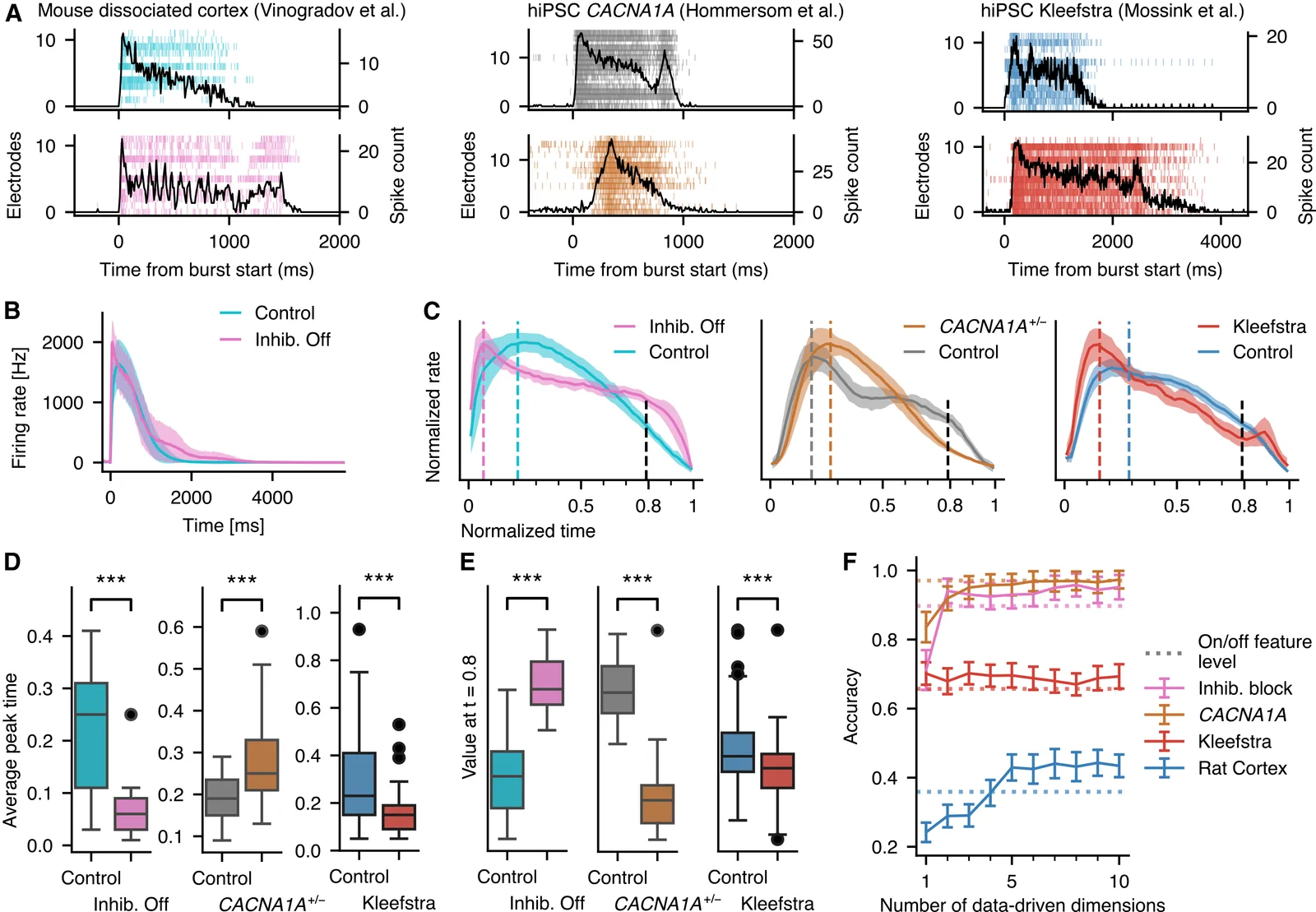
Averaged normalized burst shapes reveal altered onset and offset dynamics (A) Representative examples of raw (unnormalized) burst shapes: overlaid spike count per 10 ms bin and raster plot; control condition in the top row, same color code as in (C). (B) Averages of raw (unnormalized) burst shapes per recording are dominated by large bursts, obscuring the features evident after normalization (example of cultures with blocked inhibition). (C) Averages of normalized bursts from control cultures and cultures with blocked inhibition, *CACNA1A* haploinsufficiency, and Kleefstra syndrome. (D) Average peak timing (colored dashed lines in (C)) differs significantly from controls in all experiments, with earlier peaks in blocked inhibition and Kleefstra, and later peaks in *CACNA1A*^+/−^. (E) Amplitude near burst termination (80% of duration, black dashed line in (C)) is significantly higher for blocked inhibition, indicating abrupt termination, and reduced in *CACNA1A*^+/−^ and Kleefstra, consistent with prolonged decay dynamics. (F) The two designed features, onset (C) and offset (D), are almost as informative as the data-driven features, but are eventually surpassed as more dimensions are used. In (B) and (C), shading indicates ±3. Standard error of the mean (*N* = number of recordings, *N* = 48, *N* = 81, *N* = 390). In (D) and (E), boxplots indicate median (line), IQR (box), 1.5×IQR (whiskers), and outliers (dots); stars indicate significance with *t* test (∗*p* < 0.05, ∗∗*p* < 0.01, ∗∗∗*p* < 0.001). In (F), data are represented as mean ± standard error of the mean (*n* = 100 shuffle splits, 80–20 ratio).

Our first designed shape feature characterizes the initiation phase of bursts by the time of peak activity (Figure 4D, colored dashed lines in Figure 4C), similar to traditional approaches on raw burst shapes. Bursts in cultures with blocked inhibition reached the peak significantly faster compared to controls (inhibition off = 0.06, control = 0.21, *p* = 3.1 × 10^−7^), consistent with accelerated recruitment of network activity.^29^ *CACNA1A*^+/−^ burst shapes showed a significantly delayed peak compared to control recordings (control = 0.17, *CACNA1A*^+/−^ = 0.26, *p* = 6.4 × 10^−6^), whereas Kleefstra syndrome average burst shapes peaked earlier than controls (control = 0.28, Kleefstra = 0.15, *p* = 7.7 × 10^−14^). These findings are consistent with the analysis of absolute peak times in raw burst shapes (Figure S3) and the original studies.^10^^,^^25^

Our second designed shape feature quantifies the termination dynamics by the activity level at 80% of normalized burst duration (Figure 4E, black dashed line in Figure 4C), assessing whether bursts exhibit sustained activity with a sudden end or a prolonged decay. Cultures with blocked inhibition have significantly higher activity than controls near termination (t = 0.8, inhibition off = 0.018, control = 0.012, *p* = 4.0 × 10^−12^), indicating that inhibition smoothly terminates bursts, whereas the lack of inhibition results in a sudden drop-off in activity. *CACNA1A*^+/−^ cultures display a significantly decreased level of activity before burst termination (t = 0.8, *CACNA1A*^+/−^ = 0.008, control = 0.016, *p* = 1.1 × 10^−17^), and Kleefstra syndrome displays a similar but smaller and significant reduction (t = 0.8, Kleefstra = 0.012, control = 0.015, *p* = 3.2 × 10^−5^), indicating more continuous, prolonged decay dynamics.

The carefully designed onset and offset features contain approximately the same amount of information about culture phenotypes as the data-driven burst shape dimensions (Figure 4F). Our two designed features contain slightly more information than the two data-driven dimensions (Figure S8A). However, increasing the number of data-driven dimensions captures more information, surpassing the designed features across datasets (Figures S8B and S8C). This shows that manually designed shape features can capture sufficient information, especially in simple datasets, while data-driven shape features are reliable and scalable.

### Genetically related cultures exhibit larger burst shape similarity

Next, we asked whether genetic factors play an important role in shaping burst dynamics. To address this, we analyzed recordings from cultures with different levels of genetic relatedness and measured burst shape similarity using pairwise Wasserstein distances, comparing them to baseline distances from random pairs (Figure 5).

**Figure 5:**
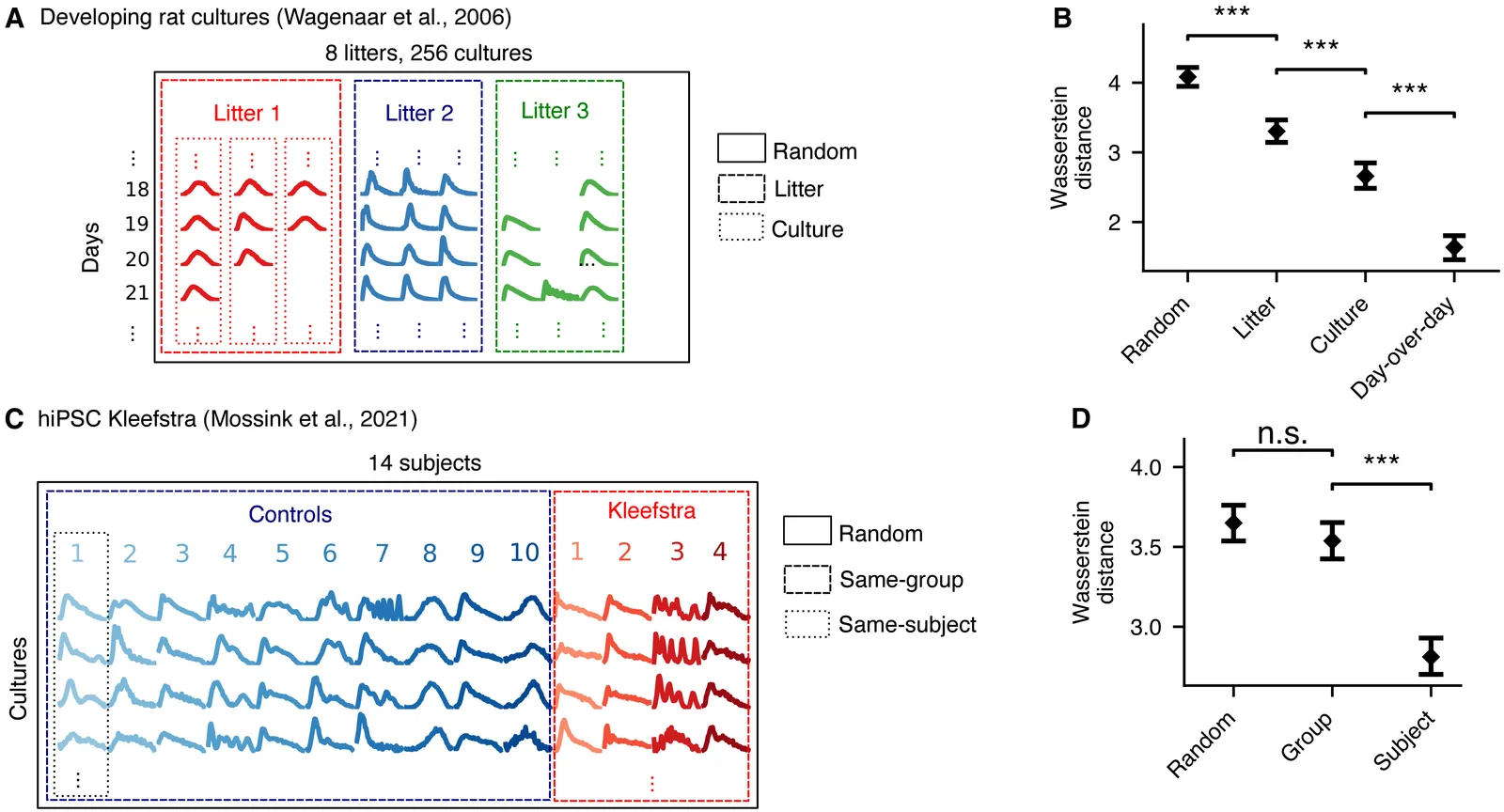
Genetically related cultures exhibit larger burst shape similarity (A) Example average burst shapes per recording of rat primary cultures during development^15^ are grouped across litters and cultures. Frames indicate comparison groups for (B). (B) Average Wasserstein distances between bursts show progressively increasing similarity: pairs from the same litter are closer than those from different litters, pairs from the same culture are closer than those from different cultures in the litter, and day-over-day variation within a culture is the smallest of all. (C) Average burst shape examples from the hiPSC disease dataset.^10^ (D) Recordings from the same subject have significantly smaller burst shape distances, while the disease group has no significant effect. Data are represented as mean and 95% confidence interval (error bars). Stars indicate significance with *t* test (∗*p* < 0.05, ∗∗*p* < 0.01, ∗∗∗*p* < 0.001).

In developing rat primary cultures,^15^ we found that burst shapes were stable across litters and throughout development. Recordings from the same litter (batches) exhibited significantly smaller burst shape distances than random comparisons (two-sided *t* test, *p* = 3.0 × 10^−12^, Figure 5B). Distances within individual cultures measured across all recording days were even smaller (*p* = 3.1 × 10^−7^), and day-to-day variability within a culture was the smallest of all (*p* = 7.1 × 10^−14^), with distances growing gradually as the gap between recordings increased (Figure S12). These findings indicate that each culture develops a characteristic burst shape that changes only slowly over time. The strong within-litter similarity suggests a genetic component, although other factors, such as seeding conditions, may also contribute. This longitudinal analysis was only feasible in the Wagenaar dataset, as the other three datasets contain a single recording per well.

In hiPSC-derived cultures,^10^ we found that burst shapes are strongly influenced by subject-specific biological identity. Recordings from the cultures derived from the same individual display significantly smaller distances than random comparisons (*p* = 4.3 × 10^−17^, Figure 5D). Interestingly, the disease status has a smaller effect on the average distance, even though it still can be readout from both the KNN-graph and hand-crafted burst-shape features (Figures 3A, 4D, and 4E). Overall, our findings indicate that burst shapes are stable across development and reflect a shared biological background, comprising genetic factors as well as shared seeding and handling.

## Discussion

Network bursts in neuronal cultures and during development *in vivo* exhibit a wide variety of profiles and durations, making their burst shapes difficult to quantify systematically. In this work, we present a data-driven approach to solving this problem, yielding a framework for extracting meaningful and practical descriptions of burst shapes. We normalize bursts to a common duration and size, enabling the pooling of variable burst shapes and the use of distance-based metrics to capture information in their profiles. This approach enhances the phenotyping of networks by using burst shape information without the need to extract specific descriptors. Thus, burst shape features contain meaningful information to distinguish between experimental conditions, such as pathological gene variants or pharmacological perturbations of neuronal activity. Moreover, we show that burst shapes also reflect a shared biological background carried by cultured neurons.

We observed distinct changes in burst shapes under pharmacological and genetic perturbations, most prominently in the onset and offset of bursts. We interpret the onset as reflecting how rapidly activity is recruited across the network, which can follow preconfigured firing sequences,^2^^,^^28^ influenced by excitability, connectivity, and synaptic transmission. Conversely, the offset reflects the processes that terminate a burst, including excitation-inhibition balance, short-term synaptic depression, and firing rate adaptation. Based on this, we hypothesize that increased excitability causes the accelerated recruitment at the onset when blocking inhibition, consistent with previous work on inhibition blockade.^29^ However, we also reveal a sudden burst termination in offset dynamics, which is far more clearly observed than in the alternative approach that relies on absolute averages and thereby obscures burst shapes. This might happen because the network lacks the inhibition to smoothly terminate bursts. Instead, bursts might collapse through exhaustion of excitation or accumulated adaptation, which was previously mechanistically modeled.^20^ *CACNA1A* haploinsufficiency leads to an elevated single-neuron excitability but to reduced synaptic transmission.^25^ Although increased intrinsic excitability would be expected to speed recruitment, the slower onset and smoother offset we observe suggest that the reduced synaptic transmission dominates at the network level. While the delayed peak was reported in the original publication, the offset change is novel, and its effect size appears to be even stronger. The earlier peak of burst shapes in Kleefstra syndrome is consistent with the upregulation of the NR1 subunit of the *N*-methyl-D-aspartate (NMDA) receptor.^14^ However, the altered burst offset remains unclear, because Kleefstra syndrome as a developmental network disease is complex, and might cause both single-cell and network-level plastic adaptations.

Although bursting robustly emerges in developing networks, variability of bursting behavior in individual cultures is known to be large.^15^ Furthermore, upon stimulation, cultures can show individually specific patterns of activity.^34^ Wagenaar et al.^15^ showed that part of this variance stems from genetic differences. Specifically, they demonstrated that “sister cultures,” cultures derived from the same litter, have lower variability compared to non-sister cultures. In our work, we reanalyzed these data and found that burst shapes are also more similar when cultures originate from the same litter. Extending these findings, when we analyzed bursts from hiPSC-derived cultures obtained from the same cell line, we found that cultures with shared biological background, including identical genomes, exhibit more similar burst shapes. This suggests a link between individual genetic differences and the variability of network burst shapes, although the precise mechanisms underlying this association remain to be determined.

### Limitations of the study

There are four main directions for future extensions of our methodology to be applicable to different scenarios. First, our approach relies on accurate burst detection, as errors in defining burst start and endpoints can misalign shapes and obscure averages. We confirmed that the key insights are preserved when using an alternative burst detection algorithm^15^^,^^35^ (Table S4; Figure S11), compared to our simple detection algorithm based on the network inter-spike interval properties.^36^ However, more complex datasets may require more sophisticated detection or alignment.^37^ Second, the embedding choice also influences the framework. While spectral embedding proved effective here, alternative methods (e.g., graph-embedding algorithms such as node2vec^38^) could capture additional structure. Third, we collapsed spiking activity across electrodes into a single time series, which facilitates application to high-throughput MEA systems but ignores spatial propagation of activity. Incorporating two-dimensional burst dynamics, especially with high-resolution recordings,^39^^,^^40^^,^^41^ may provide further insights into variability and network structure. Finally, while we demonstrate improved discrimination across four diverse datasets, the insights apply primarily to those datasets. Prospective validation across additional laboratories, MEA platforms, and screening cohorts will be needed to establish broad applicability for disease modeling and drug screening. We did not analyze the influence of sex or gender on the results.

### Conclusion

Our framework provides additional information for accurately characterizing spontaneous activity in developing neuronal cultures. Burst shape analysis enables more detailed functional phenotyping of healthy and diseased neuronal networks. Furthermore, we expect that incorporating burst shapes can contribute to the evaluation of pharmacological treatments^42^^,^^43^^,^^44^^,^^45^ and to parameter inference for mechanistic models.^5^^,^^12^^,^^46^^,^^47^^,^^48^^,^^49^

## Resource availability

### Lead contact

Requests for further information and resources should be directed to and will be fulfilled by the lead contact, Tim J. Schäfer (tim.schaefer@uni-tuebingen.de).

### Materials availability

This study did not generate new materials.

### Data and code availability

• This paper analyzes existing, publicly available data
  – Dissociated mouse cortical cultures (blocked synaptic inhibition), Vinogradov et al.,^30^ accessible at Figshare: https://doi.org/10.6084/m9.figshare.27110542.
  – Kleefstra syndrome, MELAS, and control hiPSC-derived cultures, Mossink et al.,^10^ accessible at Mendeley Data: https://doi.org/10.17632/bvt5swtc5h.1.
  – Primary rat cortical cultures (developmental time course), Wagenaar et al.,^15^ accessible at https://potterlab.bme.gatech.edu/development-data/html/daily.spont.dense.text.html.
• Data of *CACNA1A* haploinsufficiency and control hiPSC-derived cultures, Hommersom et al.,^25^ is available from the authors of the original publication upon reasonable request.
• All original code has been deposited at Zenodo and is publicly available at https://doi.org/10.5281/zenodo.21918740. It is also publicly available on GitHub at https://github.com/LevinaLab/burst-shape.
• Any additional information required to reanalyze the data reported in this paper is available from the lead contact upon request.

## Acknowledgments

This work was supported by a Sofja Kovalevskaja award from the 10.13039/100005156Alexander von Humboldt Foundation. T.J.S. received funding through the Else Kröner Medical Scientist Kolleg “ClinBrAIn: Artificial Intelligence for Clinical Brain Research,” and the 10.13039/501100001659Deutsche Forschungsgemeinschaft (DFG, 10.13039/501100001659German Research Foundation) through SFB 1690/1, A07 (project number 528760423) and under Germany’s Excellence Strategy, EXC number 2064/1 (project number 390727645). T.J.S. and E.G. thank the International Max Planck Research School for Intelligent Systems (IMPRS-IS) for support, and O.V. thanks the 10.13039/100018173International Max Planck Research School for the Mechanisms of Mental Function and Dysfunction and the 10.13039/100008662Joachim Herz Foundation for support. We acknowledge the support from the 10.13039/501100002347BMBF through the Tübingen AI Center (F.K.Z.: 01IS18039B). A.L. is a member of the Machine Learning Cluster of Excellence, EXC number 2064/1 – project number 390727645.

We thank all lab members, particularly Aaron Spieler and Elias Seiffert, for helpful discussions and their support. We also thank Farzin Negahbani for helpful discussions. A big thank you also to Marina Hommersom and Nina Doorn for sharing their data and helpful discussions.

## Author contributions

Conceptualization, T.J.S., E.G., A.L., and O.V.; methodology, T.J.S., E.G., A.L., and O.V.; investigation, T.J.S., E.G., and P.S.-B.; writing – original draft, T.J.S., E.G., A.L., and O.V.; writing – review and editing, T.J.S., E.G., A.L., and O.V.; funding acquisition, A.L.; resources, A.L. and O.V.; supervision, A.L. and O.V.

## Declaration of interests

The authors declare no competing interests.

## Declaration of generative AI and AI-assisted technologies in the writing process

During the preparation of this work, the author(s) used Claude, GitHub Copilot, and ChatGPT to support coding, and used Claude, ChatGPT, Gemini, and Perplexity to support writing. After using this tool or service, the author(s) reviewed and edited the content as needed and take full responsibility for the content of the publication.

## STAR★Methods

### Key resources table

**Table:** 

REAGENT or RESOURCE	SOURCE	IDENTIFIER
**Deposited data**		

MEA recordings, dissociated mouse cortical cultures (blocked synaptic inhibition)	Vinogradov et al.^30^; Figshare	Figshare: https://doi.org/10.6084/m9.figshare.27110542
MEA recordings, *CACNA1A* haploinsufficiency and control hiPSC-derived cultures	Hommersom et al.^25^	Available from the authors of the original publication upon reasonable request
MEA recordings, Kleefstra syndrome, MELAS, and control hiPSC-derived cultures	Mossink et al.^10^; Mendeley Data	Mendeley Data: https://doi.org/10.17632/bvt5swtc5h.1
MEA recordings, primary rat cortical cultures (developmental time course)	Wagenaar et al.^15^	https://potterlab.bme.gatech.edu/development-data/html/daily.spont.dense.text.html

**Software and algorithms**		

Custom analysis code for this study	This paper; Zenodo	Zenodo: https://doi.org/10.5281/zenodo.21918740

### Experimental model and study participant details

This study is a reanalysis of previously published neural culture recordings. Details on species, strain/line, genotype, age, culturing, and ethical oversight are as reported in the original publications cited for each dataset and are summarized in the Datasets paragraph. We did not analyze the influence of sex or gender.

#### Datasets

We utilized four distinct micro-electrode array (MEA) datasets from neural cultures, including both rat and human induced pluripotent stem cell (hiPSC)-derived cultures. Three of these datasets were publicly available, and one^25^ was requested from the authors of the original publication. For all datasets, we used the processed spiking data, which consists of the timestamped spike trains for each electrode. Each recording corresponds to a single timepoint per well and was treated as an independent sample; recordings from different days *in vitro* (DIV) were not pooled.

##### Blocked inhibition

This dataset was published by Vinogradov et al.^30^ and consists of recordings from dissociated mouse cortical neurons (data is available online at https://figshare.com/articles/dataset/MEA_recordings/27110542). The neurons were cultured on 24-well MEA plates, each well containing 12 gold electrodes. For two batches, the spontaneous network activity was recorded at 17 and 18 days *in vitro* (batches 1 and 2). To block synaptic inhibition, 40 *μ*M of bicuculline was added to 10 of the 24 wells in each batch, resulting in *N* = 20 recordings with blocked inhibition and *N* = 28 recordings as control (*N* = 48 total). Following a 10-min stabilization period, network activity was recorded for 20 min. While the original study did not discuss burst shapes, this dataset is well-suited for our analysis due to its systematic modulation of network activity.

##### CACNA1A haploinsufficiency

This dataset was provided by Hommersom et al.^25^ and consists of human induced pluripotent stem cell-derived cultures. The control line was modified with CRISPR/Cas9 to generate *CACNA1A* haploinsufficiency and validated for pluripotency markers and trilineage differentiation potential. The original study does not report whether the cell lines were tested for mycoplasma contamination. The cultures were recorded at DIV 42 for 5 min using 48-well MEA plates, each well containing 16 electrodes. The data comprises four batches, with 24 recordings from *CACNA1A*^+/−^ cultures and 57 recordings from controls (recordings that did not display bursting activity were excluded from the analysis). The original study reported a slightly delayed peak time of bursts from *CACNA1A* haploinsufficiency compared to controls, which suggests a systematic burst shape difference relevant to our investigation.

##### Kleefstra syndrome

This dataset was published by Mossink et al.^10^ and consists of hiPSC cultures derived from skin fibroblasts of control and disease patients. The dataset includes cultures from four Kleefstra patients, five healthy controls, and five isogenic controls (modified patient cells). We group the controls with *N* = 287 recordings (one per well) and compare against the *N* = 58 Kleefstra recordings. The study also contained three MELAS (Mitochondrial Encephalopathy, Lactic Acidosis, and Stroke-like episodes) syndrome patients, which we excluded from our analysis because the original study reported that MELAS burst shapes were very similar to those of controls. In contrast, Kleefstra bursts were reported to have differing onset dynamics, making them suitable for our investigation and simplifying the classification to a binary problem of Kleefstra versus control networks. Recordings span DIV 27 to 35, the stable burst-parameter window identified by Mossink et al.,^10^ and each well in each batch contributes a single recording. The cell lines have been authenticated per the original study’s supplementary information (short tandem repeat profiling, single nucleotide polymorphism array analysis, or karyotyping). It is not explicitly stated whether testing for mycoplasma contamination was performed.

##### Primary neuronal rat cultures

This dataset was published by Wagenaar et al.^15^ and consists of anterior cortical neural cultures from Wistar rat embryos, extracted on day 18 of gestation. Cells from one litter were combined into a single batch, yielding eight batches (litters) in total. Each batch is thus a mixture of several embryos with shared genetics. The cultures were recorded for 30 min without a prior stabilization period. We selected the data between days 7 and 35 *in vitro* for our analysis, resulting for the eight batches (1–8) in 74, 137, 102, 28, 18, 40, 28, and 24 recordings, *N* = 451 in total. While focusing on the developmental trajectories of burst summary statistics, the original study also included simple features of intra-burst dynamics, such as onset and offset durations. They reported that burst durations decreased over development, particularly the burst onset duration, as shown in Figure 6 of the original study.

### Method details

#### Preprocessing

##### Burst identification

We use a method of combining spike times from all electrodes and detecting bursts previously suggested by Bakkum et al.^35^ and adapted to various settings.^20^^,^^50^ First, we concatenated the spike times from all recording units (electrodes) into a single network-wide spike train. We then used a detection algorithm to find the start and end of bursts based on the inter-spike interval (ISI).^36^^,^^51^^,^^52^ A burst was defined to start when the ISI fell below a predefined threshold ISI_start_ and to end when the following ISI exceeded a second threshold ISI_end_. While these thresholds were often identical, they could be set independently of each other.

To filter for meaningful network events, we applied additional criteria. A detected burst was accepted as a burst only if it (1) had a minimum burst duration BD_min_, (2) a minimum inter-burst interval IBI_min_, and (3) a minimum burst size (number of spikes) BS_min_. We also applied optional filters for a minimum burst length and a minimum firing rate. In some instances, a 4 ms smoothing kernel was also applied to mitigate the influence of fluctuations in very short bursts. The chosen parameters are shown in Table S2.

To test robustness, we implemented and ran the analysis with an additional burst detection algorithm. This algorithm first detects burst events on individual electrodes based on the ISI.^36^^,^^51^^,^^52^ We chose a simple deterministic mapping of parameters from our burst detection to this algorithm based on the number of electrodes *N*: ISI threshold ${I\hat{S}I}_{\text{start/end}}={ISI}_{\text{start/end}}\cdot \sqrt{N}$ and minimum burst size ${\hat{BS}}_{\min}={BS}_{\min}/N$ (all other parameters are the same). Then, for detecting network events, we threshold simultaneously active electrodes,^29^^,^^52^^,^^53^ and choose a 20% threshold.^51^^,^^54^ The rationale for this threshold is that we believe the high-activity time frame to be important for burst shapes.

Table S4 shows that the algorithms mostly agree on the burst detection. Even for the dataset with the lowest agreement (83.9% overlap time), the average burst shapes look very similar (Figure S11A). Running it for all datasets shows that the key result is preserved, that augmenting traditional features with burst shape features captures significantly more information about network phenotypes (Figures S11B and S11C).

We also tested how sensitive burst detection is to a perturbation of the main parameter: the threshold of the inter-spike interval ISI_start∕end_. We systematically changed ISI_start∕end_ from −50% to +50%, finding that for most perturbations the detection remains stable (Table S5). For small perturbations up to 20% there is almost a one-to-one match. Only for extreme perturbations of −50% or +50% does the burst existence agreement (F1 value) and the overlapping time (Dice index) drop more than 10% and only in a few datasets. We estimate that this range within a 10% change of the F1 value and Dice index is safe, because the alternative burst detection had larger deviations and still preserved our key insights (Table S4; Figure S11).

##### Burst review

After running the automated detection algorithm, we manually reviewed the burst detection using a custom interactive tool (Figure S1). This tool allowed us to efficiently examine the quality of burst detection across a large number of recordings. The tool overlays the detected bursts on traces of the firing rate and raster plots, with the option to zoom in for closer inspection. If the initial parameters did not accurately capture the network bursts, we could adjust the burst detection parameters until an acceptable level of accuracy was achieved. On average, between 88.8% and 98.9% of electrodes participated in these bursts, depending on the dataset (Figure S13), confirming that they are network events.

#### Spectral embedding with Wasserstein distance

##### Normalization

To compare bursts using the Wasserstein distance, we normalized the bursts in both time and amplitude, thereby isolating their shape feature. We normalized duration to a length of 1, discretizing it into 50 time bins and calculating the firing rate for each bin from the spike times. Finally, we normalized each burst shape by its integral (the sum of all bins) to ensure a total integral of 1. These steps resulted in a standardized burst shape with unit duration and unit integral, which was then used in further analysis.

##### Wasserstein distance

To quantify the similarity between burst shapes, we chose the Wasserstein distance metric (also called Earth Mover’s distance). This metric was selected because it is robust to small shifts, assigning a small distance to similar burst shapes and a large distance to dissimilar ones. For two bursts *b*_1_ and *b*_2_, the one-dimensional Wasserstein distance *d*(., .) can be calculated from their cumulative sums:

(Equation 1) $$d\left(b_{1},b_{2}\right)=\underset{j}{\sum }\left|\overset{j}{\underset{i=0}{\sum }}b_{1}^{i}-\overset{j}{\underset{i=0}{\sum }}b_{2}^{i}\right|,$$

where $b_{n}^{i}$ is the value of burst *n* at bin *i*.

We compared alternative distance metrics (KL-divergence, Euclidean, correlation) and found that Wasserstein distance gave the most consistent improvement, although KL-divergence might be a viable alternative for specific datasets and burst shapes (Figures S9 and S10).

##### K-nearest neighbors (KNN) graph

To capture the relationships between individual burst shapes, we constructed a KNN graph, which connects each burst shape with its *K* most similar burst shapes. The pairwise distances between all burst shapes were computed using the Wasserstein distance. The number of nearest neighbors *K* was selected such that each node was connected to approximately 1% of all nodes (similar to the recommendation for t-SNE perplexity by Kobak and Berens^55^), resulting in *K* between 21 and 150 for our datasets. This KNN graph served as the basis for both KNN clustering and spectral embedding.

##### Spectral embedding

To obtain a low-dimensional embedding from the KNN graph, we performed spectral embedding. First, we constructed the symmetric adjacency matrix $A_{\text{sym}}=\frac{1}{2}\left(A+A^{T}\right)$ from the unsymmetric adjacency matrix *A* of the KNN graph. Next, we computed the Graph Laplacian L = *D* − *A*_sym_, where *D* is the degree matrix such that each row (and column) of *L* sums up to zero. Then, we used the eigenvectors of *L* corresponding to the smallest non-zero eigenvalues as the low-dimensional coordinates, as they best preserve the connectivity structure of the underlying graph.

#### Classification of recordings

To understand which burst features contain biologically meaningful information, we classified recordings with various models and feature sets. We employed two primary classification strategies. First, we used KNN-based classification to leverage the rich information within individual burst shapes, aggregating this information to the recording level. Second, we applied XGBoost to classify recordings based on summary features, including traditional, shape, and combined feature sets. For all classification tasks, we evaluated model performance using balanced accuracy and a repeated stratified shuffle split (100 times, 80–20 training-testing split) for robust cross-validation.

##### KNN prediction from individual burst shapes

To leverage the rich information present in individual burst shapes, we performed a KNN-based classification. To prevent data leakage, we first ensured that no individual bursts from test-set recordings were included in the training set. Then, for each of these ‘test bursts’, we identified its *K* nearest neighbors among the ‘training bursts’. We predicted the class of the ‘test burst’ with a voting scheme based on these neighbors, which belonged to a training recording with a known class label. To account for class imbalances and recording-specific biases, we weighted the vote of each neighbor. The weight was calculated as the inverse of the number of bursts in its recording $N_{r_{j}}$ and the inverse of the number of recordings in its class $N_{c_{j}}$.

The class vote vector **v**_*i*_ for an individual burst was computed as

(Equation 2) $$v_{i}=\overset{K}{\underset{j=1}{\sum }}\frac{1}{N_{r_{j}}}\cdot \frac{1}{N_{c_{j}}}\cdot b_{ij}$$

where **b**_*ij*_ is a one-hot vector indicating the class label of the *j*-th neighbor of test burst *i*. To obtain a prediction for a test recording, we averaged the vote vectors **v**_*i*_ across all its constituent bursts. The class with the highest average vote was assigned as the predicted label for that recording.

##### XGBoost prediction from summary statistics

To assess the predictive power of shape features relative to traditional burst features, we trained XGBoost on three sets of recording-level summary statistics: shape features, traditional features, and a combined feature set.

The *traditional burst features*, used as a baseline for prediction accuracy, were derived from commonly used burst metrics.^11^

• Mean firing rate (MFR): average of the culture’s firing rate
• Burst spike rate (BSR): average of the bursts’ firing rates
• Network burst rate (NBR): number of bursts divided by recording duration
• Network burst duration (NBD): average of burst durations
• Percentage of Random Spikes (PRS): percentage of spikes not assigned to bursts

We excluded some features used by van Hugte et al.^11^ (mean burst rate (MBR), burst duration (BD), and high-frequency bursts (HFB)) because they rely on single-channel bursts and high-frequency bursts, which were not computed in our analysis.

Our *burst shape features* were based on the spectral embedding, the low-dimensional projection of the KNN graph. For each recording, we averaged the individual burst shape locations in two dimensions. This yielded two shape summary features per recording (spectral embedding dimensions 1 and 2). While more or fewer dimensions than two were possible, we always chose two dimensions, the same as in the visualizations. The *combined feature set* is obtained by combining the five traditional and the two burst shape features.

For all XGBoost models, we employed a nested cross-validation approach with an outer (testing) and inner loop (validation). The outer loop consisted of a repeated stratified shuffle split (100 times, 80–20 split) for testing (same as in KNN classification). The inner loop optimized the XGBoost hyperparameters via a grid search in a 5-fold cross-validation. We report the balanced accuracy on the test set to evaluate the predictive power of each feature set.

##### Feature importance with Shapley values

To quantify the relative contribution of traditional and shape features, we computed Shapley values^33^ (feature importance) for the XGBoost model trained on the combined feature set. Shapley values are a robust method from cooperative game theory for fairly distributing the ‘payout’ (in this case, the model’s prediction) among the ‘players’ (the features). This approach is used in machine learning to quantify the importance of features while accounting for complex interactions. A particularly useful property of Shapley values is their additivity, which allowed us to calculate the total importance of the traditional feature set and directly compare it to that of the shape features.

### Quantification and statistical analysis

Statistical details are reported in the respective figure legends and main text. Unless stated otherwise, *N* is the number of recordings and values are mean ± standard error of the mean. Sample sizes were fixed by the number of recordings available in each dataset, so they were not determined by a prior power analysis. Significance was defined as *p* < 0.05 and we used asterisks to represent significance levels: ∗: *p* < 0.05, ∗∗: *p* < 0.01, ∗∗∗: *p* < 0.001. To ensure reproducibility, all randomized procedures, e.g., repeated train-validation-test partitioning, used fixed random seeds.

#### Exclusion criteria

Recordings without any detected bursts were excluded. In the hiPSC disease dataset,^10^ the three MELAS patients were excluded because the original study reported MELAS burst shapes to be indistinguishable from controls. In the rat developmental dataset,^15^ the analysis was restricted to recordings between 7 and 35 days *in vitro*. No further recording-level exclusions were applied.

#### Comparison of model accuracies

For comparing model accuracies (Figures 3B–3D), we recognized that standard methods such as a *t* test lead to inflated significance because accuracies from cross-validation are interdependent due to the reuse of data.^31^^,^^32^ Therefore, to robustly compare model accuracies, we used the correction for the standard error of the mean ${\hat{s}}_{d}^{2}={\hat{\sigma }}^{2}\left(\frac{1}{n}+\frac{n_{te}}{n_{tr}}\right)$, where *n* is the number of trials, *n*_*te*_ is test set size, and *n*_*tr*_ is training set size. With our cross-validation parameters (*n* = 100 and $\frac{n_{te}}{n_{tr}}=\frac{1}{4}$), we computed a paired two-sided *t* test. To test the overall statement across datasets, we computed a meta-analysis using a random-effects model and applied a Wald test.

#### Feature comparison

Differences between manually designed features (Figures 4D and 4E) were compared with an unpaired *t* test.

#### Distance comparison

We analyzed the distances between recordings (Figures 5B and 5D) using a multi-step process. First, we calculated the pairwise distances between recordings by averaging the distances between their constituent individual burst shapes. We then filtered the pairwise distances based on specific criteria, for example, including all distances (“random”) or only distances between recordings from the same subject (“subject”). To balance the dataset and avoid falsely inflating sample size, we computed a single average distance for each recording by averaging all outgoing distances. Finally, we used the two-sided *t* test to determine if there were significant differences in these average distances.

## References

[1] Wu M.W., Kourdougli N., Portera-Cailliau C. (2024). Network state transitions during cortical development. Nat. Rev. Neurosci..

[2] Xie W., Wittig J.H., Chapeton J.I., El-Kalliny M., Jackson S.N., Inati S.K., Zaghloul K.A. (2024). Neuronal sequences in population bursts encode information in human cortex. Nature.

[3] Soriano J., Rodríguez Martínez M., Tlusty T., Moses E. (2008). Development of input connections in neural cultures. Proc. Natl. Acad. Sci. USA.

[4] Penn Y., Segal M., Moses E. (2016). Network synchronization in hippocampal neurons. Proc. Natl. Acad. Sci. USA.

[5] Gao R., Deistler M., Schulz A., Gonçalves P.J., Macke J.H. (2024). Deep inverse modeling reveals dynamic-dependent invariances in neural circuit mechanisms. bioRxiv.

[6] Giugliano M., Darbon P., Arsiero M., Lüscher H.R., Streit J. (2004). Single-neuron discharge properties and network activity in dissociated cultures of neocortex. J. Neurophysiol..

[7] Van Pelt J., Corner M.A., Wolters P.S., Rutten W.L.C., Ramakers G.J.A. (2004). Longterm stability and developmental changes in spontaneous network burst firing patterns in dissociated rat cerebral cortex cell cultures on multielectrode arrays. Neurosci. Lett..

[8] Segev R., Shapira Y., Benveniste M., Ben-Jacob E. (2001). Observations and modeling of synchronized bursting in two-dimensional neural networks. Phys. Rev. E.

[9] Hyvärinen T., Hyysalo A., Kapucu F.E., Aarnos L., Vinogradov A., Eglen S.J., Ylä-Outinen L., Narkilahti S. (2019). Functional characterization of human pluripotent stem cell-derived cortical networks differentiated on laminin-521 substrate: comparison to rat cortical cultures. Sci. Rep..

[10] Mossink B., Verboven A.H.A., van Hugte E.J.H., Klein Gunnewiek T.M., Parodi G., Linda K., Schoenmaker C., Kleefstra T., Kozicz T., Van Bokhoven H. (2021). Human neuronal networks on micro-electrode arrays are a highly robust tool to study disease-specific genotype-phenotype correlations in vitro. Stem Cell Rep..

[11] van Hugte E.J.H., Lewerissa E.I., Wu K.M., Scheefhals N., Parodi G., van Voorst T.W., Puvogel S., Kogo N., Keller J.M., Frega M. (2023). SCN1a-deficient excitatory neuronal networks display mutation-specific phenotypes. Brain.

[12] Doorn N., Voogd E.J.H.F., Levers M.R., van Putten M.J.A.M., Frega M. (2024). Breaking the burst: Unveiling mechanisms behind fragmented network bursts in patient-derived neurons. Stem Cell Rep..

[13] Hedrich U.B.S., Liautard C., Kirschenbaum D., Pofahl M., Lavigne J., Liu Y., Theiss S., Slotta J., Escayg A., Dihné M. (2014). Impaired action potential initiation in GABAergic interneurons causes hyperexcitable networks in an epileptic mouse model carrying a human NaV1.1 mutation. J. Neurosci..

[14] Frega M., Linda K., Keller J.M., Gümüş-Akay G., Mossink B., Van Rhijn J.R., Negwer M., Klein Gunnewiek T., Foreman K., Kompier N. (2019). Neuronal network dysfunction in a model for Kleefstra syndrome mediated by enhanced NMDAR signaling. Nat. Commun..

[15] Wagenaar D.A., Pine J., Potter S.M. (2006). An extremely rich repertoire of bursting patterns during the development of cortical cultures. BMC Neurosci..

[16] Charlesworth P., Cotterill E., Morton A., Grant S.G.N., Eglen S.J. (2015). Quantitative differences in developmental profiles of spontaneous activity in cortical and hippocampal cultures. Neural Dev..

[17] Obien M.E.J., Deligkaris K., Bullmann T., Bakkum D.J., Frey U. (2014). Revealing neuronal function through microelectrode array recordings. Front. Neurosci..

[18] Cerina M., Piastra M.C., Frega M. (2023). The potential of in vitro neuronal networks cultured on micro electrode arrays for biomedical research. Prog. Biomed. Eng..

[19] Soriano J. (2023). Neuronal cultures: Exploring biophysics, complex systems, and medicine in a dish. Biophysica.

[20] Sukenik N., Vinogradov O., Weinreb E., Segal M., Levina A., Moses E. (2021). Neuronal circuits overcome imbalance in excitation and inhibition by adjusting connection numbers. Proc. Natl. Acad. Sci. USA.

[21] Estévez-Priego E., Moreno-Fina M., Monni E., Kokaia Z., Soriano J., Tornero D. (2023). Long-term calcium imaging reveals functional development in hiPSC-derived cultures comparable to human but not rat primary cultures. Stem Cell Rep..

[22] Okujeni S., Kandler S., Egert U. (2017). Mesoscale architecture shapes initiation and richness of spontaneous network activity. J. Neurosci..

[23] Suresh J., Radojicic M., Pesce L.L., Bhansali A., Wang J., Tryba A.K., Marks J.D., Van Drongelen W. (2016). Network burst activity in hippocampal neuronal cultures: The role of synaptic and intrinsic currents. J. Neurophysiol..

[24] Kim D., Kang H., Nam Y. (2020). Compact 256-channel multi-well microelectrode array system for in vitro neuropharmacology test. Lab Chip.

[25] Hommersom M.P., Doorn N., Puvogel S., Lewerissa E.I., Mordelt A., Ciptasari U., Kampshoff F., Dillen L., Van Beusekom E., Oudakker A. (2025). *CACNA1A* haploinsufficiency leads to reduced synaptic function and increased intrinsic excitability. Brain.

[26] Gigante G., Mattia M., Del Giudice P. (2007). Diverse Population-Bursting Modes of Adapting Spiking Neurons. Phys. Rev. Lett..

[27] Baltz T., Herzog A., Voigt T. (2011). Slow oscillating population activity in developing cortical networks: Models and experimental results. J. Neurophysiol..

[28] Van Der Molen T., Spaeth A., Chini M., Hernandez S., Kaurala G.A., Schweiger H.E., Duncan C., McKenna S., Geng J., Lim M. (2026). Preconfigured neuronal firing sequences in human brain organoids. Nat. Neurosci..

[29] Eytan D., Marom S. (2006). Dynamics and Effective Topology Underlying Synchronization in Networks of Cortical Neurons. J. Neurosci..

[30] Vinogradov O., Giannakakis E., Buendía V., Uysal B., Ron S., Weinreb E., Schwarz N., Lerche H., Moses E., Levina A. (2024). Effective excitability captures network dynamics across development and phenotypes. bioRxiv.

[31] Nadeau C., Bengio Y. (1999). Inference for the generalization error. Adv. Neural Inf. Process. Syst..

[32] Benavoli A., Corani G., Demšar J., Zaffalon M. (2017). Time for a change: a tutorial for comparing multiple classifiers through bayesian analysis. J. Mach. Learn. Res..

[33] Lundberg S.M., Lee S.I. (2017). A unified approach to interpreting model predictions. Adv. Neural Inf. Process. Syst..

[34] Pasquale V., Martinoia S., Chiappalone M. (2017). Stimulation triggers endogenous activity patterns in cultured cortical networks. Sci. Rep..

[35] Bakkum D.J., Radivojevic M., Frey U., Franke F., Hierlemann A., Takahashi H. (2013). Parameters for burst detection. Front. Comput. Neurosci..

[36] Cotterill E., Charlesworth P., Thomas C.W., Paulsen O., Eglen S.J. (2016). A comparison of computational methods for detecting bursts in neuronal spike trains and their application to human stem cell-derived neuronal networks. J. Neurophysiol..

[37] Hernandes V., Heuvelmans A.M., Gualtieri V., Meijer D.H., van Woerden G.M., Greplova E. (2024). autoMEA: machine learning-based burst detection for multi-electrode array datasets. Front. Neurosci..

[38] Grover A., Leskovec J. (2016). Proceedings of the 22nd ACM SIGKDD International Conference on Knowledge Discovery and Data Mining.

[39] Haroush N., Marom S. (2019). Inhibition increases response variability and reduces stimulus discrimination in random networks of cortical neurons. Sci. Rep..

[40] Orlandi J.G., Soriano J., Alvarez-Lacalle E., Teller S., Casademunt J. (2013). Noise focusing and the emergence of coherent activity in neuronal cultures. Nat. Phys..

[41] Yuan X., Schröter M., Obien M.E.J., Fiscella M., Gong W., Kikuchi T., Odawara A., Noji S., Suzuki I., Takahashi J. (2020). Versatile live-cell activity analysis platform for characterization of neuronal dynamics at single-cell and network level. Nat. Commun..

[42] Sundberg M., Pinson H., Smith R.S., Winden K.D., Venugopal P., Tai D.J.C., Gusella J.F., Talkowski M.E., Walsh C.A., Tegmark M., Sahin M. (2021). 16p11.2 deletion is associated with hyperactivation of human iPSC-derived dopaminergic neuron networks and is rescued by RHOA inhibition in vitro. Nat. Commun..

[43] Parodi G., Zanini G., Collo L., Impollonia R., Cervetto C., Frega M., Chiappalone M., Martinoia S. (2024). In vitro electrophysiological drug testing on neuronal networks derived from human induced pluripotent stem cells. Stem Cell Res. Ther..

[44] Fofie C.K., Granja-Vazquez R., Truong V., Walsh P., Price T., Biswas S., Dussor G., Pancrazio J., Kolber B. (2025). Profiling human iPSC-derived sensory neurons for analgesic drug screening using a multi-electrode array. Cell Rep. Methods.

[45] Raabe F.J., Popovic D., Vetter C., Fischer L.E., Hasanaj G., Karslı B., Schäfer T.J., Almeida V., Atella A., Gagliardi M. (2026). Bridging the scales via personalized cellular modeling and deep phenotyping in schizophrenia. JAMA Psychiatry.

[46] Masquelier T., Deco G. (2013). Network Bursting Dynamics in Excitatory Cortical Neuron Cultures Results from the Combination of Different Adaptive Mechanism. PLoS One.

[47] Cohen D., Segal M. (2011). Network bursts in hippocampal microcultures are terminated by exhaustion of vesicle pools. J. Neurophysiol..

[48] Yamamoto H., Spitzner F.P., Takemuro T., Buendía V., Murota H., Morante C., Konno T., Sato S., Hirano-Iwata A., Levina A. (2023). Modular architecture facilitates noise-driven control of synchrony in neuronal networks. Sci. Adv..

[49] Doorn N., Van Putten M.J.A.M., Frega M. (2025). Automated inference of disease mechanisms in patient-hiPSC-derived neuronal networks. Commun. Biol..

[50] Sit T.P.H., Feord R.C., Dunn A.W.E., Chabros J., Oluigbo D., Smith H.H., Burn L., Chang E., Boschi A., Yuan Y. (2024). MEA-NAP: A flexible network analysis pipeline for neuronal 2D and 3D organoid multielectrode recordings. Cell Rep. Methods.

[51] Bologna L.L., Pasquale V., Garofalo M., Gandolfo M., Baljon P.L., Maccione A., Martinoia S., Chiappalone M. (2010). Investigating neuronal activity by SPYCODE multi-channel data analyzer. Neural Netw..

[52] Chiappalone M., Novellino A., Vajda I., Vato A., Martinoia S., Van Pelt J. (2005). Burst detection algorithms for the analysis of spatio-temporal patterns in cortical networks of neurons. Neurocomputing.

[53] Van Pelt J., Vajda I., Wolters P.S., Corner M.A., Ramakers G.J.A. (2005). Dynamics and plasticity in developing neuronal networks in vitro. Prog. Brain Res..

[54] Hu M., Frega M., Tolner E.A., Van Den Maagdenberg A.M.J.M., Frimat J.P., Le Feber J. (2022). MEA-ToolBox: An Open Source Toolbox for Standardized Analysis of Multi-Electrode Array Data. Neuroinformatics.

[55] Kobak D., Berens P. (2019). The art of using t-SNE for single-cell transcriptomics. Nat. Commun..

