# Supplementary material for: Data-driven burst shape analysis for functional phenotyping of neuronal cultures
Source: iScience. 2026 Sep 21;29(10):117499. doi: 10.1016/j.isci.2026.117499 (PMC13631671; doi:10.1016/j.isci.2026.117499)
Supplement: Supplementary file 1 [file mmc1.pdf]

iScience, Volume *29*

Supplemental information

Data-driven burst shape analysis for functional phenotyping of neuronal cultures

Tim J. Schäfer, Emmanouil Giannakakis, Paul Schmidt-Barbo, Anna Levina, and Oleg Vinogradov

Supplemental information

Dataset	#cultures	#bursts	Time (min)	Total time (min)
Blocked inhibition	48	8571	20	960
CACNA1A	81	2114	5	405
hiPSC disease	390	9350	20	7800
Developing rat	451	11914	60	27060

Table S1: **Summary statistics of datasets used in the analysis.**

Parameter	Blocked inhibition	*CACNA1A*	Developing rat	hiPSC disease
$\mathrm{ISI}_{\mathrm{start}}$	20	20	5	100
$\mathrm{ISI}_{\mathrm{end}}$	20	20	5	50
$\mathrm{BD}_{\mathrm{min}}$	50	50	40	100
$\mathrm{IBI}_{\mathrm{min}}$	100	100	40	500
$\mathrm{BS}_{\mathrm{min}}$	100	100	50	50
min_length	30	30	30	30
min_firing_rate	-	-	3162	-
smoothing_kernel	-	-	4	-

Table S2: **Network burst detection parameters.**
For blocked-inhibition (bicuculline) [S1], *CACNA1A* haploinsufficiency [S2], developing embryo rat cultures [S3], and hiPSC disease recordings [S4], we detect the bursts based on the parameters given in the table in milliseconds.
ISI – inter-spike interval, $\mathrm{ISI}_{\mathrm{start}}$ – maximal ISI, $\mathrm{ISI}_{\mathrm{end}}$ – minimal ISI during the burst, $\mathrm{BD}_{\mathrm{min}}$ – minimal duration of the burst, $\mathrm{IBI}_{\mathrm{min}}$ – minimal inter-burst interval, $\mathrm{BS}_{\mathrm{min}}$ – minimal number of spikes in burst, min_length – minimal length of the burst, min_firing_rate – minimal average firing rate, smoothing_kernel – length of smoothing kernel.

Dataset	Random	Summary Features<br>Traditional	 Shape	 Combined	Individual<br>burst shapes
Blocked inhibition	0.5	0.750	0.937	0.949* #	**0.981*** #**
CACNA1A	0.5	0.887	0.917	0.944 †	**0.998**** #
hiPSC disease (Kleefstra)	0.5	0.785	0.677	**0.828** †	0.726 ‡
Developing rat	0.125	0.535	0.289	**0.608** †	0.559

Table S3: **Mean classification accuracies.**
Balanced accuracies for the cross-validated classification task across datasets. Traditional (baseline, highlighted in blue): XGBoost on summary features, shape: XGBoost on shape features, combined: both feature sets. KNN classification uses individual burst shapes aggregated to the recording level. Stars indicate frequentist comparison to the baseline (corrected for data interdependence): *: $p < 0.05$, **: $p < 0.01$, ***: $p < 0.001$. Daggers indicate Bayesian comparison to baseline: † moderate evidence (posterior $P > .7$), ‡ strong ($P > .9$), # very strong evidence ($P > .99$). Bold: best model.

Dataset	#bursts<br>(population/electrode)	Corresponding bursts<br>(F1 value)	Overlapping time<br>(Burst Dice index)
Blocked inhibition	8571 / 9056	.988	.968
CACNA1A	2114 / 2179	.991	.939
hiPSC disease	8599 / 9456	.936	.900
Developing rat	11915 / 13221	.948	.839

Table S4: **Network burst detection algorithms agree.**
1) The algorithms (the population-based detection vs. the alternative electrode-based algorithm) deliver a comparable number of bursts. 2) In all datasets, more than 90% of bursts have a corresponding burst in the other algorithm (F1 value). 3) The times strongly agree in all datasets, reported as Burst Dice index (the percentage of overlap, $= \frac{2 \cdot (T_1 \cap T_2)}{T_1 + T_2}$), with detected burst time T_i of algorithm i). This indicates that the alternative algorithm produces a set of bursts that has almost a one-to-one match with our population-based algorithm with potential small shifts in start and end.

	Block. Inhib.			*CACNA1A*			hiPSC Kleefstra			Rat cortex		
ΔISI	N	F1	Dice	N	F1	Dice	N	F1	Dice	N	F1	Dice
-50%	8943	.9973	.9475	2124	.9991	.8848	8065	.9642	.8403	13844	.9273	.8078
-20%	8668	.9997	.9859	2114	.9998	.9632	8479	.9916	.9516	12429	.9792	.9392
-10%	8616	.9999	.9935	2114	.9998	.9820	8536	.9956	.9760	12147	.9904	.9711
-5%	8596	.9999	.9969	2114	1.0000	.9904	8574	.9982	.9883	12026	.9954	.9852
0%	8571	1.0000	1.0000	2114	1.0000	1.0000	8599	1.0000	1.0000	11915	1.0000	1.0000
$+5\%$	8555	.9999	.9971	2114	1.0000	.9925	8621	.9982	.9891	11797	.9949	.9858
$+10\%$	8536	.9999	.9947	2116	.9995	.9838	8649	.9964	.9792	11690	.9905	.9730
$+20\%$	8507	.9998	.9897	2117	.9993	.9677	8701	.9932	.9596	11451	.9803	.9456
$+50\%$	8434	.9994	.9779	2119	.9981	.9271	8821	.9850	.9058	10682	.9457	.8735

Table S5: **Sensitivity of the population-based burst detection algorithm to the $\mathrm{ISI}_{\mathrm{start/end}}$ threshold.**
The detection is compared against the unperturbed run on the number of detected bursts N, burst existence (F1 value), and the percentage of overlapping time (Dice index $= \frac{2\cdot(T_1 \cap T_2)}{T_1+T_2}$), with detected burst time T_i of algorithm i).
Block. Inhib.: High degree of agreement with any of these perturbations. *CACNA1A*: Bursts match one-to-one (F1 value), but timing changes up to 13% (Dice index). hiPSC Kleefstra: highly stable unless $\mathrm{ISI}_{\mathrm{start/end}}$ is changed by -50%, +50%, then timing changes by up to 16% (Dice index). Rat cortex: Gradual degradation when perturbing $\mathrm{ISI}_{\mathrm{start/end}}$, confirming that this is the most heterogeneous dataset.

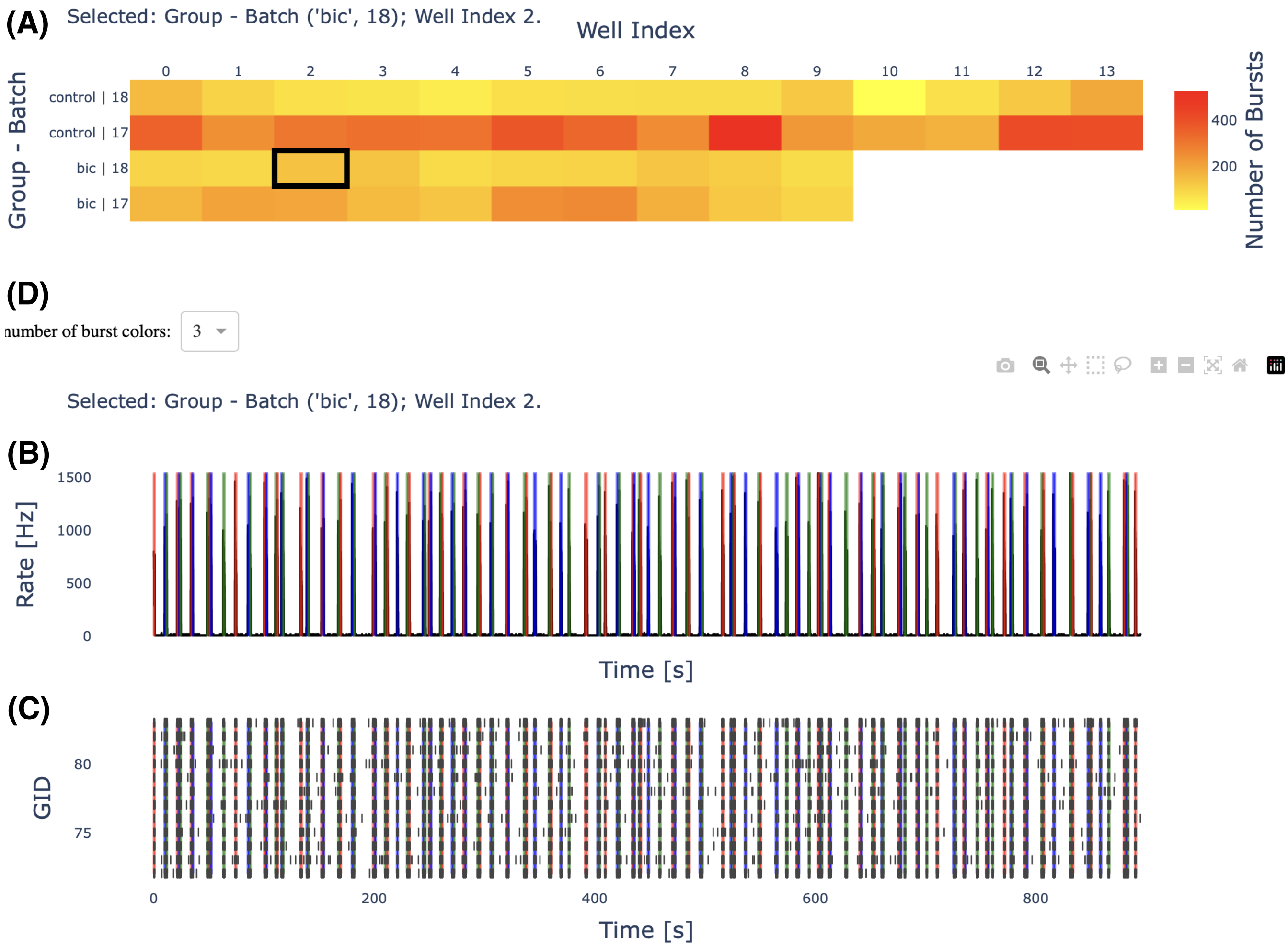

Figure S1: **Interactive tools enable efficient processing of large amounts of data (part 1).** Screenshot of the tool for the blocked inhibition data [S1]. These tools can be deployed offline or online (https://github.com/LevinaLab/burst-shape).

Burst detection review: This tool enables fast and detailed evaluation of the burst detection algorithm. We use it to improve the burst detection parameters iteratively. Note that there is neither manual adjustment of burst start/end nor manual rejection/inclusion; the tool is purely for quality control.

(A) Overview of all recordings. Rows and columns are the index: Here, it is `Well Index` for columns and `Group-Batch` for rows. The color indicates the number of bursts detected in the recording. Clicking on one of the recordings opens up the firing rate **(B)** and raster plot **(C)** for that recording. The firing rate and raster plot have a synchronized time axis. The detected bursts are highlighted with a colored background, where the number of colors can be adjusted for improved visualization **(D)**. To inspect details of burst detection, the user can zoom in by selecting a time frame.

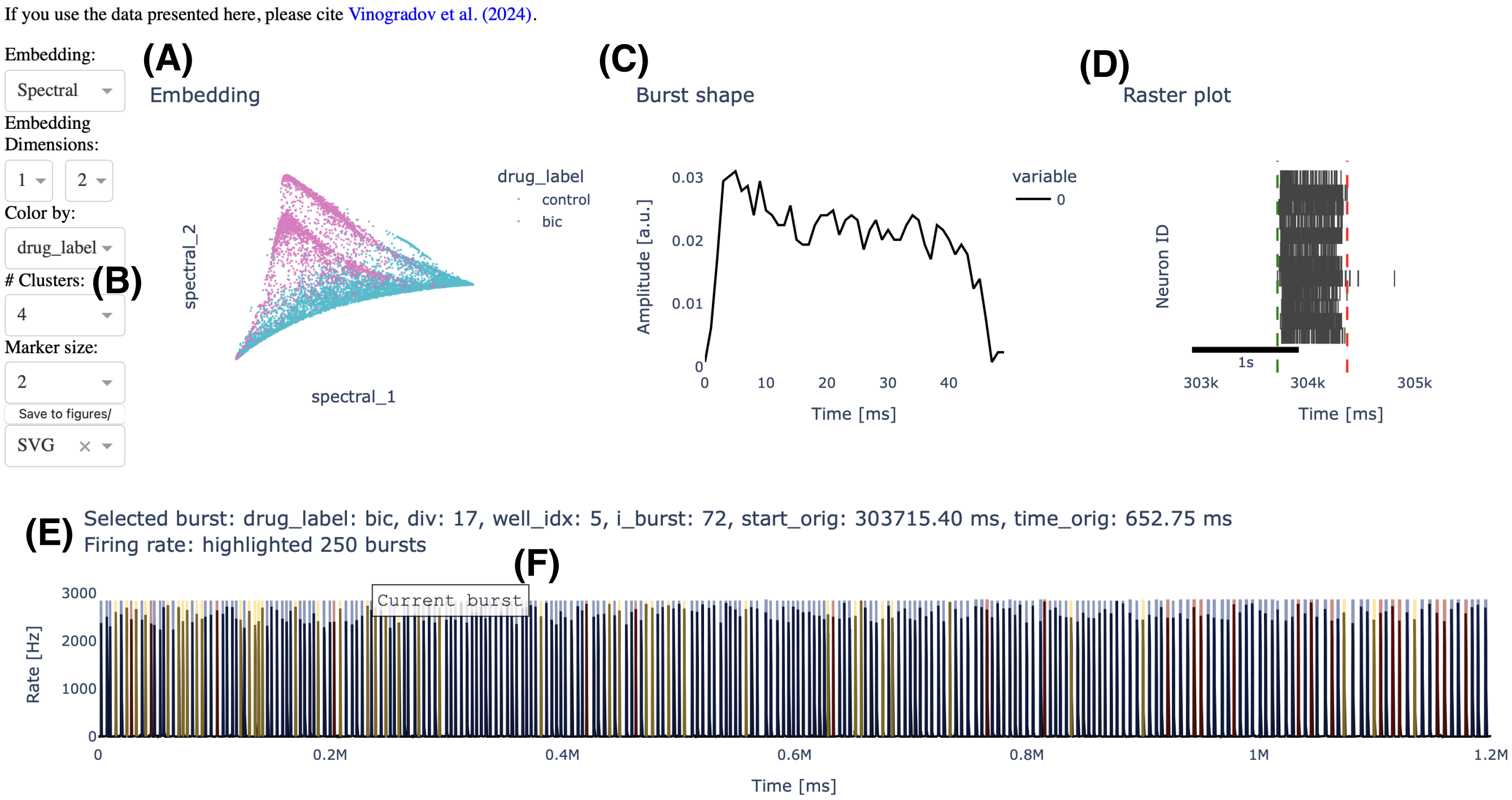

Figure S2: **Interactive tools enable efficient processing of large amounts of data (part 2).** Screenshot of the tool for the blocked inhibition data [S1]. These tools can be deployed offline or online (https://github.com/LevinaLab/burst-shape).

Burst shape embedding visualization: This tool enables fast interactive exploration of the embeddings of burst shapes.

(A) The embedding of individual burst shapes can be explored interactively by zooming in and selecting and deselecting groups. **(B)** Options to change the embedding type (Spectral, PCA, or t-SNE), the plotted dimensions (up to 20th dimension), the color-code criterion (batch, firing rate, burst duration, spectral clusters, etc.), the number of spectral clusters (relevant if color=cluster), and the marker size. When selecting (clicking) one of the nodes (burst shapes) in the embedding (A), the application displays the burst shape **(C)**, the raster plot **(D)**, and the associated recordings' firing rate **(E)**, with the selected burst highlighted **(F)**. It is possible to directly switch to another burst from the same recording by clicking on it.

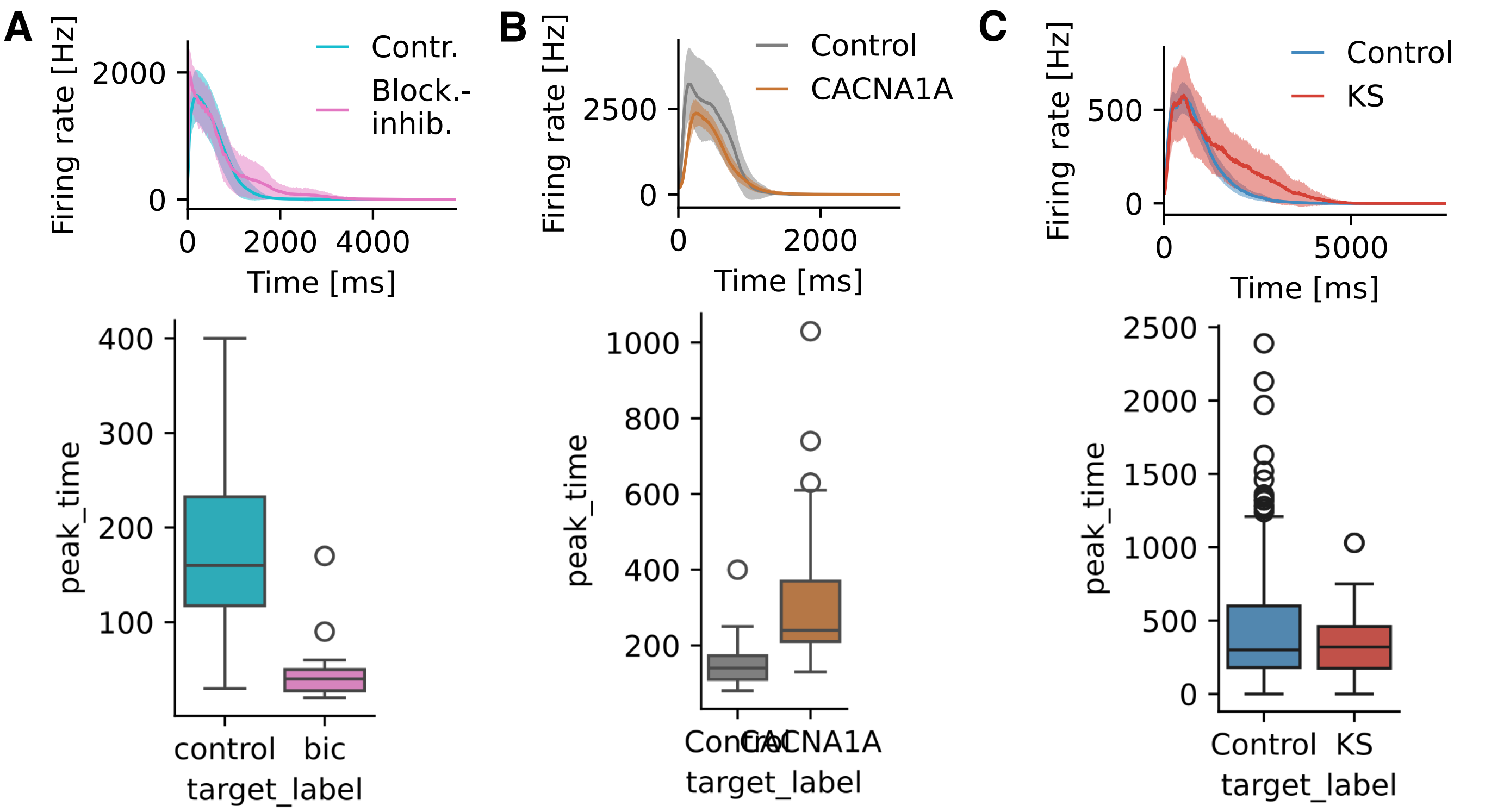

Figure S3: **Raw shape differences between groups.**
Averaging raw burst shapes enables comparison of onset dynamics, but makes it impossible to quantify burst termination dynamics.
(A) Blocked inhibition recordings burst shapes peak significantly earlier than controls (bic=46.5 ms, control=175.4 ms, p=1.1e-17).
(B) *CACNA1A* recordings burst shapes peak significantly later than controls (control=155.0 ms, *CACNA1A*=298.6 ms, p=2.4e-7).
(C) Kleefstra recording burst shapes peak significantly earlier than controls (control=445.5 ms, Kleefstra=344.1 ms, p=5.7e-3).
In the top row, shading indicates $\pm$ 3·standard error of the mean (N=number of recordings, N=48, N=81, N=390). In the bottom row, box plots indicate median (line), IQR (box), $1.5\times$IQR (whiskers), and outliers (dots); p-values from t-test.

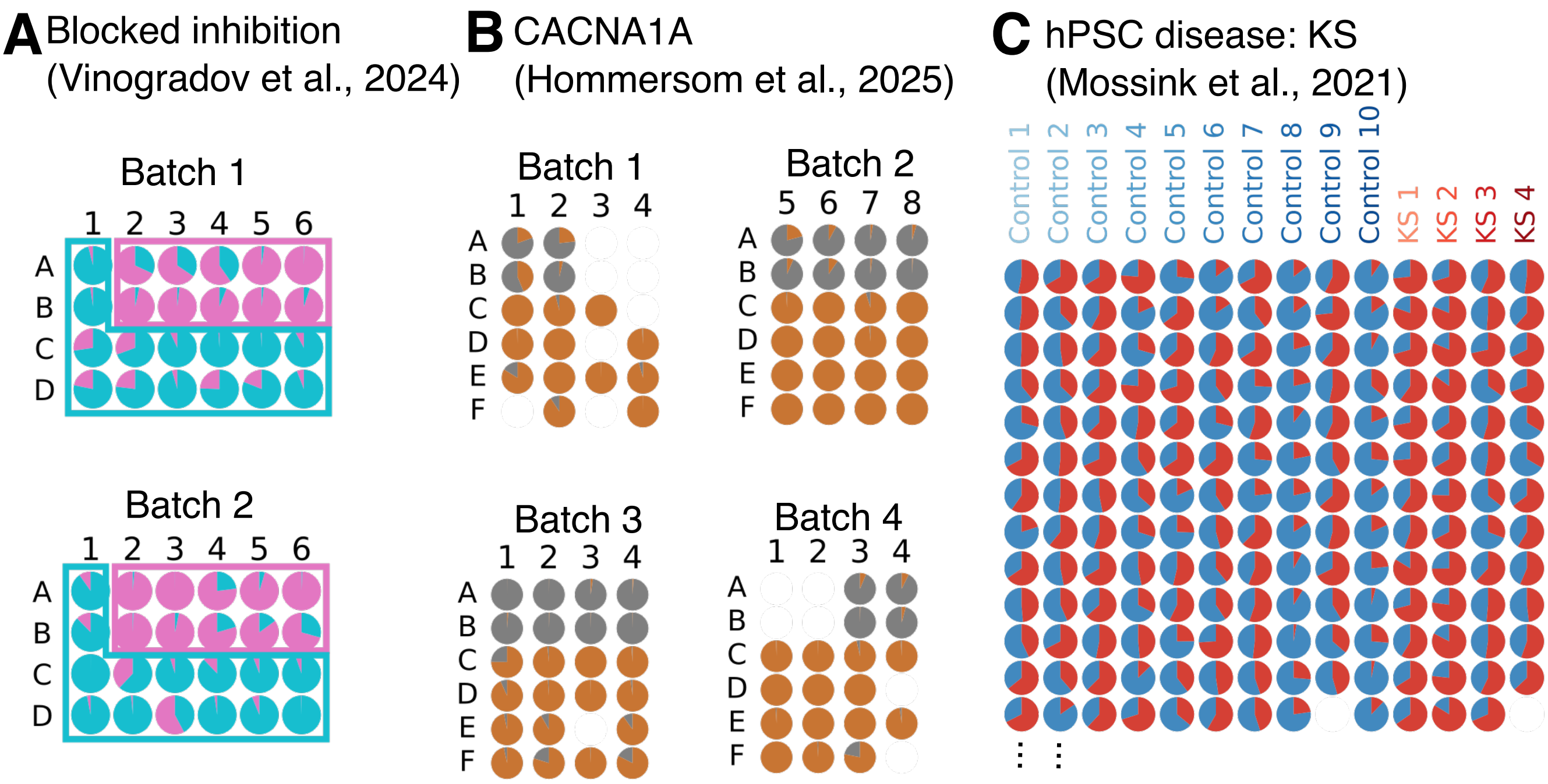

Figure S4: **K-nearest neighbor classification predictions (part 1).**
Predictions are the *votes* from the K nearest neighbors weighted by class and number of bursts per recording. Each pie chart represents the share of these votes for one recording, indicating the confidence of the prediction. These predictions are obtained using leave-one-out cross-validation, in contrast to the main text, which employs a stricter cross-validation with a repeated 80-20 split.
(A) Blocked inhibition [S1] predictions plotted in the plate layout in which the cultures were recorded. Rectangles indicate the true labels. **(B)** *CACNA1A* haploinsufficiency vs control [S2] predictions plotted in the plate layout in which the cultures were recorded (*CACNA1A*$^{+/-}$ is located in rows A and B; controls are located in rows C-F). **(C)** Subset of hiPSC disease recordings [S4] (only Control vs Kleefstra) predictions sorted by subject.

A Developing rat
(Wagenaar et al., 2006)

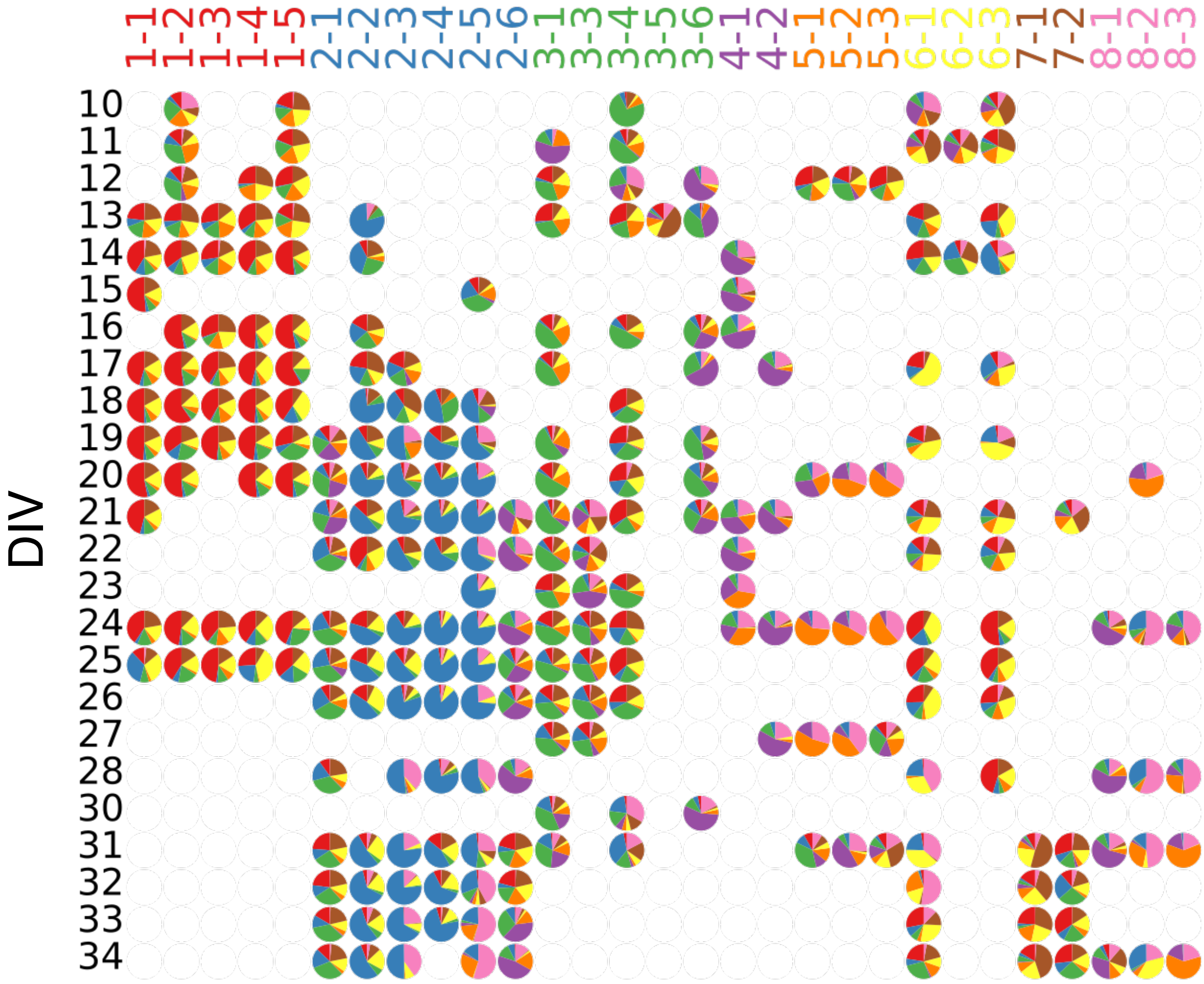

Figure S5: **K-nearest neighbor classification predictions (part 2).**
Predictions are the *votes* from the K nearest neighbors weighted by class and number of bursts per recording. Each pie chart represents the share of these votes for one recording, indicating the confidence of the prediction. These predictions are obtained using leave-one-out cross-validation, in contrast to the main text, which employs a stricter cross-validation with a repeated 80-20 split.
Columns are the days *in vitro* (DIV). Thus, developmental trajectories are from top to bottom.
(A) Developing rat primary [S3] recordings predictions. Batches (true labels) are the colors of the columns. Column labels are `[batch] - [index of culture within batch]`.

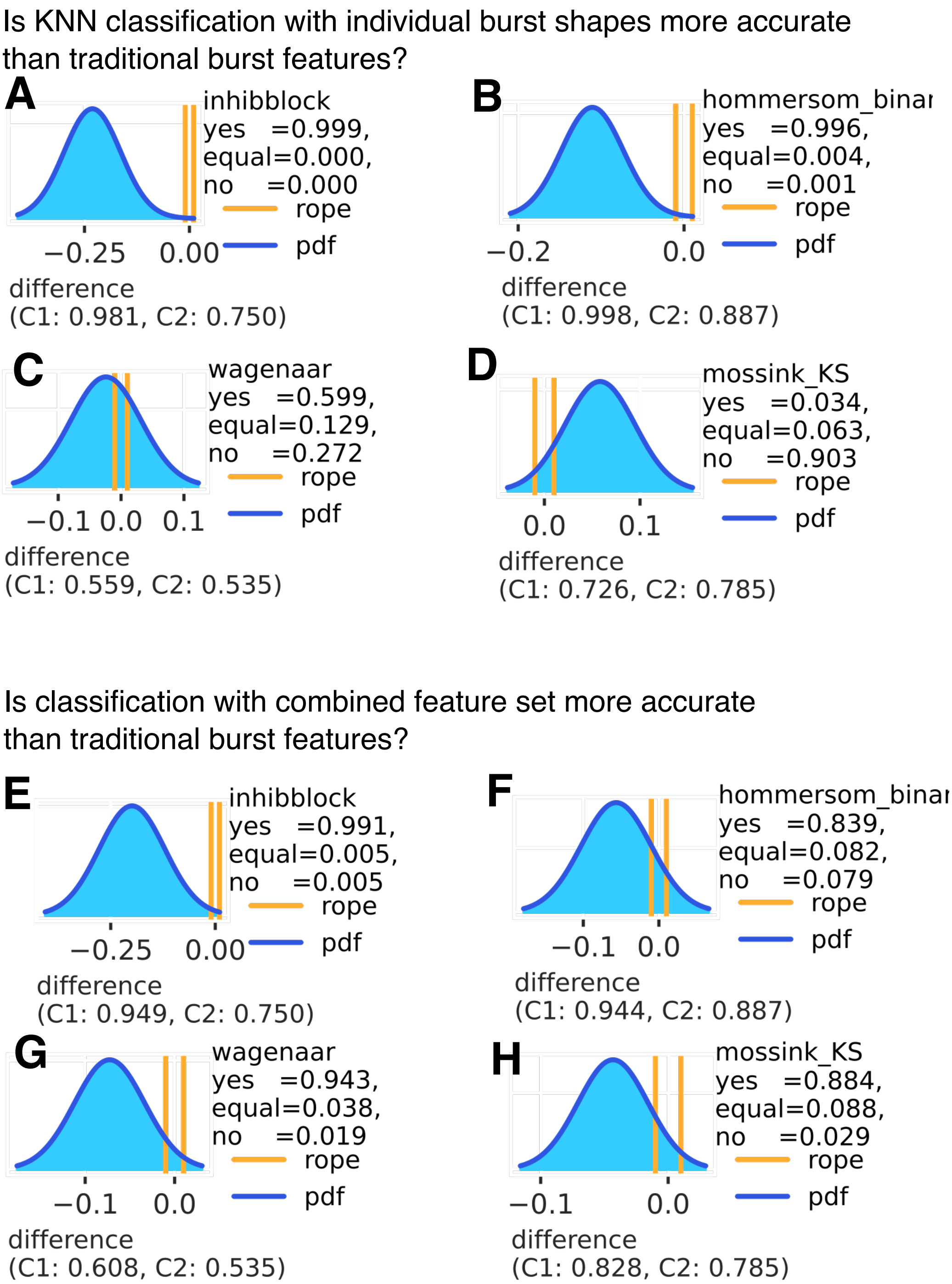

Figure S6: **Bayesian comparison of classification accuracies.**
Bayesian comparison using the baycomp toolbox (`https://github.com/janezd/baycomp`), with a region of practical equivalence (rope) defined as ±1 % accuracy. The output is the posterior probability distribution that one method is more accurate than the other (see above the *yes* and *no* probability).
(A-D) comparing KNN classification on individual burst shapes against traditional burst shape features.
(E-H) comparing combined features (shape + traditional) set against traditional burst features.

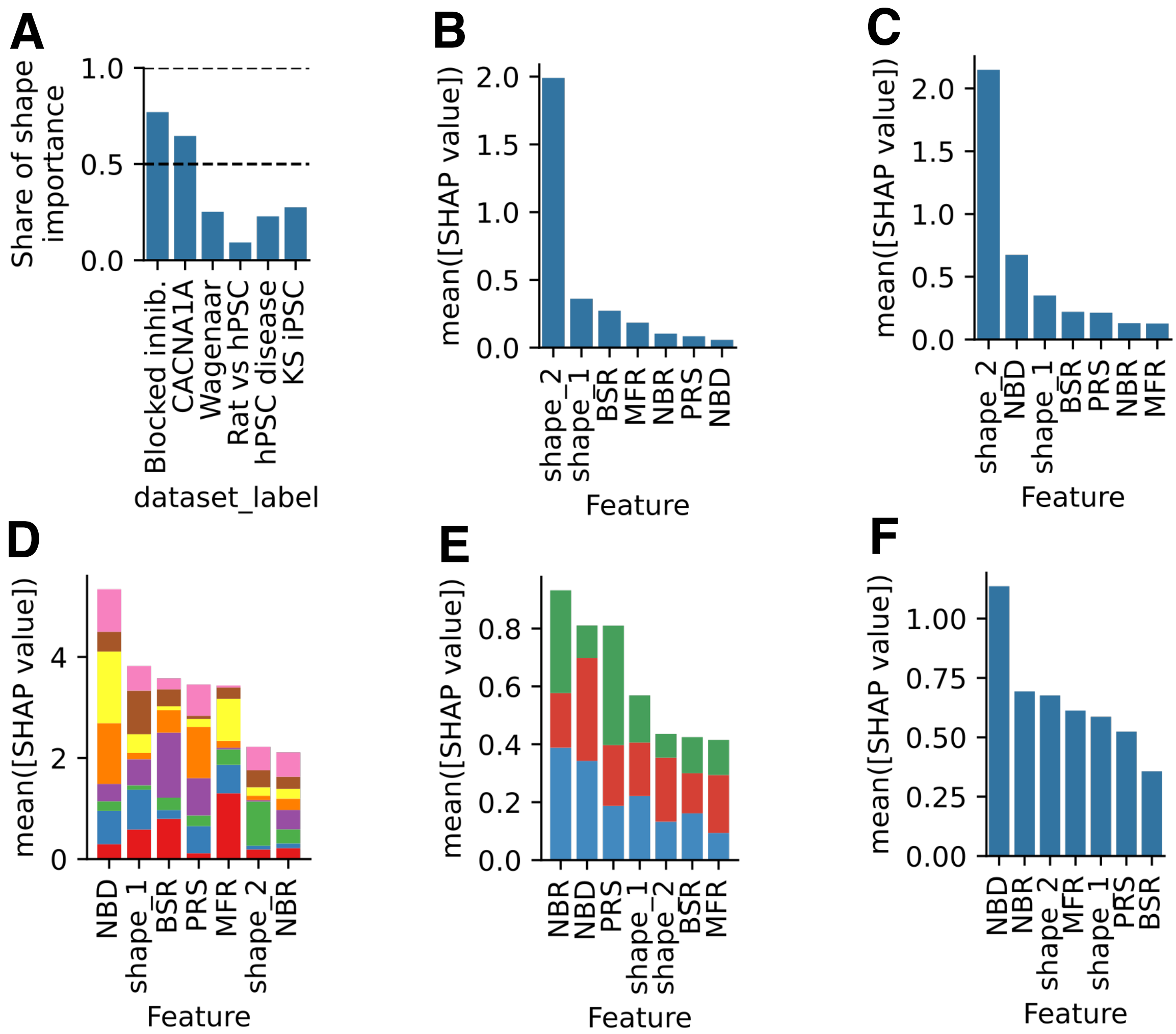

Figure S7: **Feature importance measured with Shapley values.**
(A) Summed up feature importance from both shape features (Shapley values are additive).
(B-G) Feature importance from fitting XGBoost with the combined feature set. Order indicates order of importance. Colors are group colors if more than two groups are present.
Datasets:
(B) Blocked-inhibition [S1] **(C)** *CACNA1A* [S2] **(D)** Developing rat primary [S3] (colors represent batches) **(E)** Mossink: Kleefstra, MELAS, and Control [S4] (colors represent disease groups) **(F)** Mossink: only Kleefstra and Control [S4].

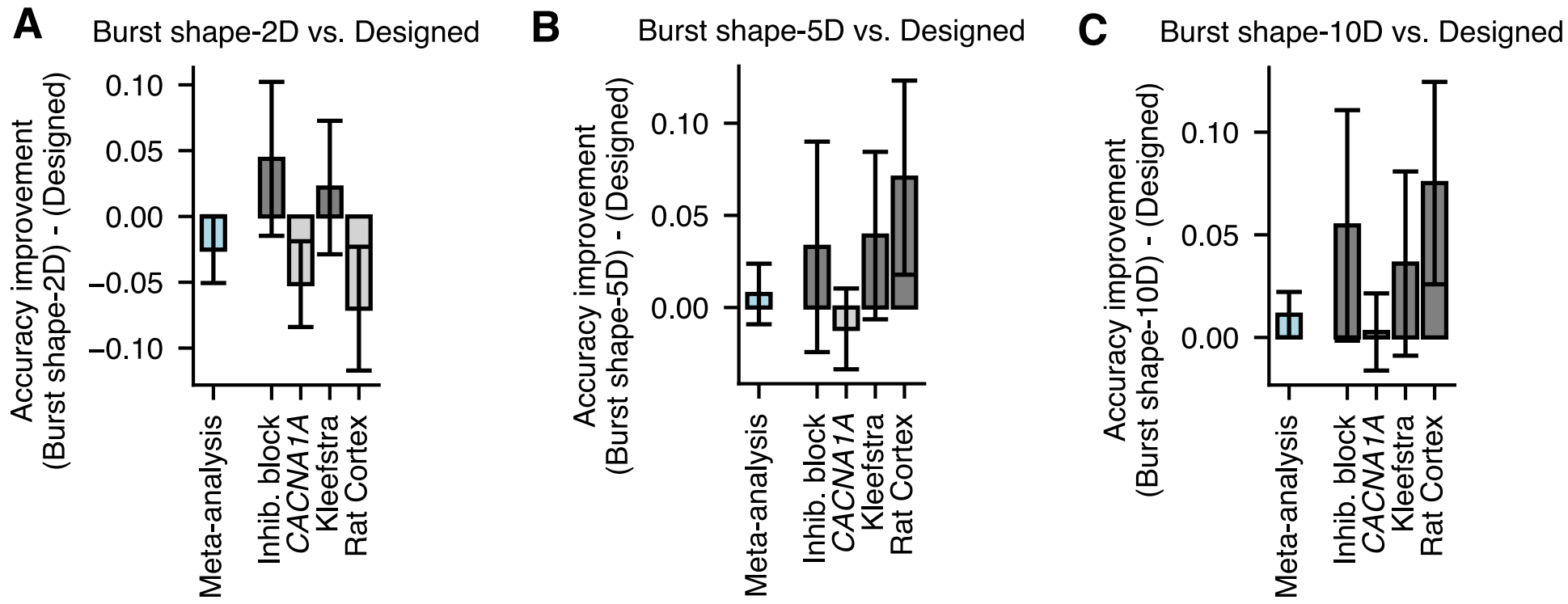

Figure S8: **Designed features (onset and offset) are almost as good as data-driven features from spectral embedding. (A)** The two designed features perform slightly better than the two spectral embedding dimensions. **(B)** Five spectral data-driven features are better than the designed features. **(C)** Ten data-driven features are better than the designed features across all datasets.

Data are represented as mean $\pm$ standard error of the mean (n=100 shuffle splits, 80–20 ratio). Stars indicate significance with Wald test (*: $p < 0.05$, **: $p < 0.01$, ***: $p < 0.001$); meta-analysis (hypothesis stated on top of each panel) used a random-effects model.

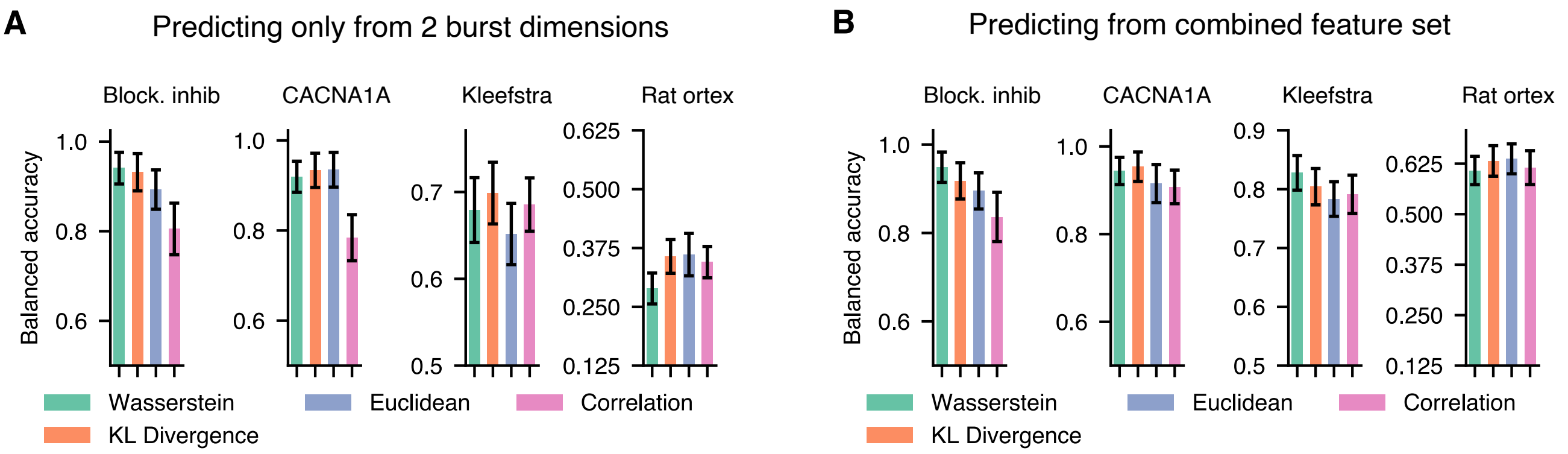

Figure S9: **Alternative metrics: Accuracies are comparable.**

For each alternative metric, we computed the accuracy of predicting based on two burst shape dimensions **(A)** and based on the combined feature set **(B)**. Even though Wasserstein distance appears to be among the better choices for a distance metric, especially for the combined features (B), most of the differences fall within the standard error of the mean (error bars).

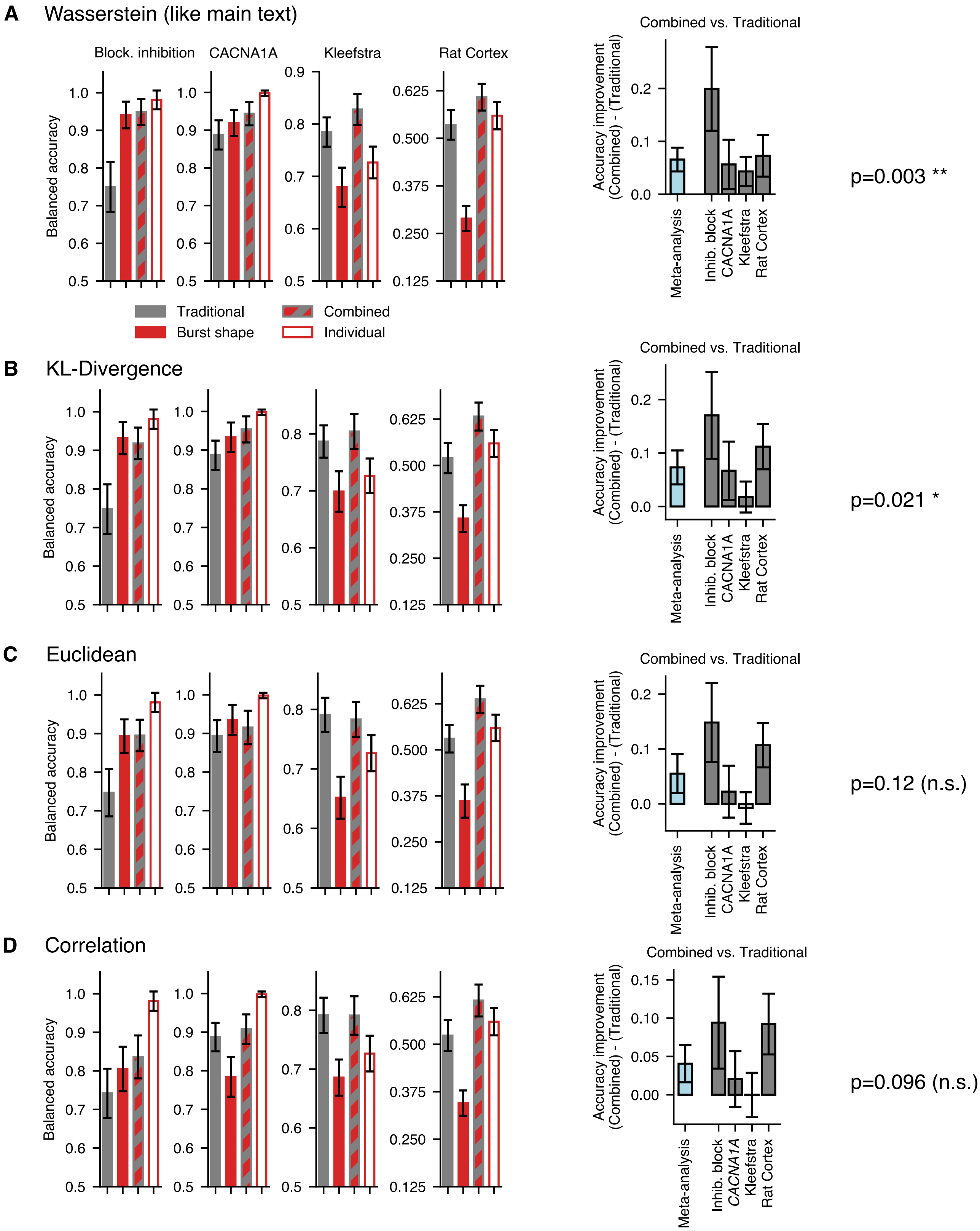

Figure S10: **Alternative metrics: Improvement over traditional feature baseline is best with Wasserstein metric.**

We recomputed the result of Figure 3A,B (same as in this Figure **(A)**) for the alternative metrics: **(B)** Kullback-Leibler (KL) Divergence, **(C)** Euclidean, and **(D)** Correlation distance. The Wasserstein distance provides a consistent improvement over the baseline across all datasets, making it more significant in the meta-analysis (p=0.003) than the KL-Divergence (p=0.021), Euclidean distance (p=0.12), or Correlation metric (p=0.096).

Data are represented as mean ± standard error of the mean (n=100 shuffle splits, 80–20 ratio). p-values with Wald test; meta-analysis (hypothesis stated on top of each panel) used a random-effects model.

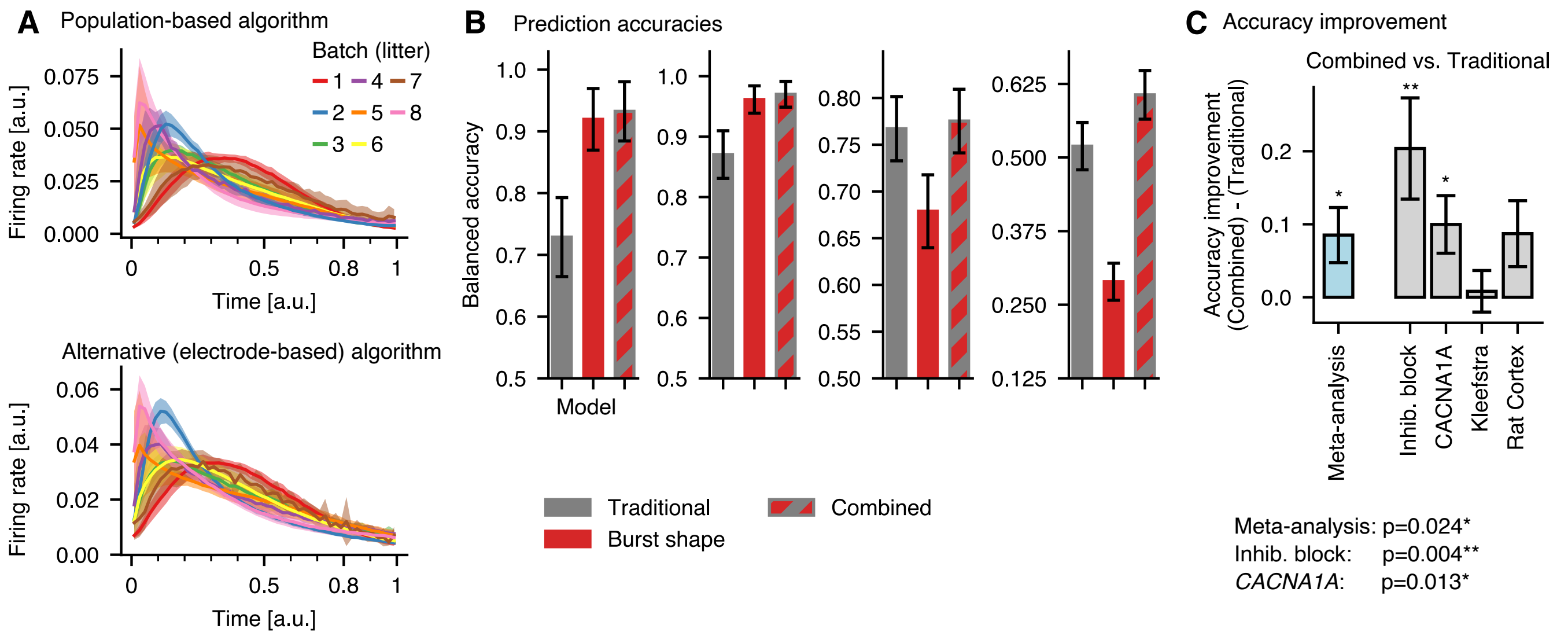

Figure S11: **Alternative (electrode-based) burst detection reproduces key result that burst shape features capture network phenotype.**
(A) The burst shapes averaged per batch (litter) are similar, and importantly, the group means remain tight (shading indicates $\pm$ 3$\cdot$standard error of the mean). **(B,C)** Classification accuracies based on the alternative (electrode-based) algorithm show that the combined feature set (burst shape + traditional) captures significantly more information on network phenotype than traditional features alone.
In (A), shading indicates $\pm$ 3$\cdot$standard error of the mean. In (B) and (C), data are represented as mean $\pm$ standard error of the mean (n=100 shuffle splits, 80–20 ratio). In (C), stars indicate significance with Wald test (*: $p < 0.05$, **: $p < 0.01$, ***: $p < 0.001$); meta-analysis (hypothesis stated on top of each panel) used a random-effects model.

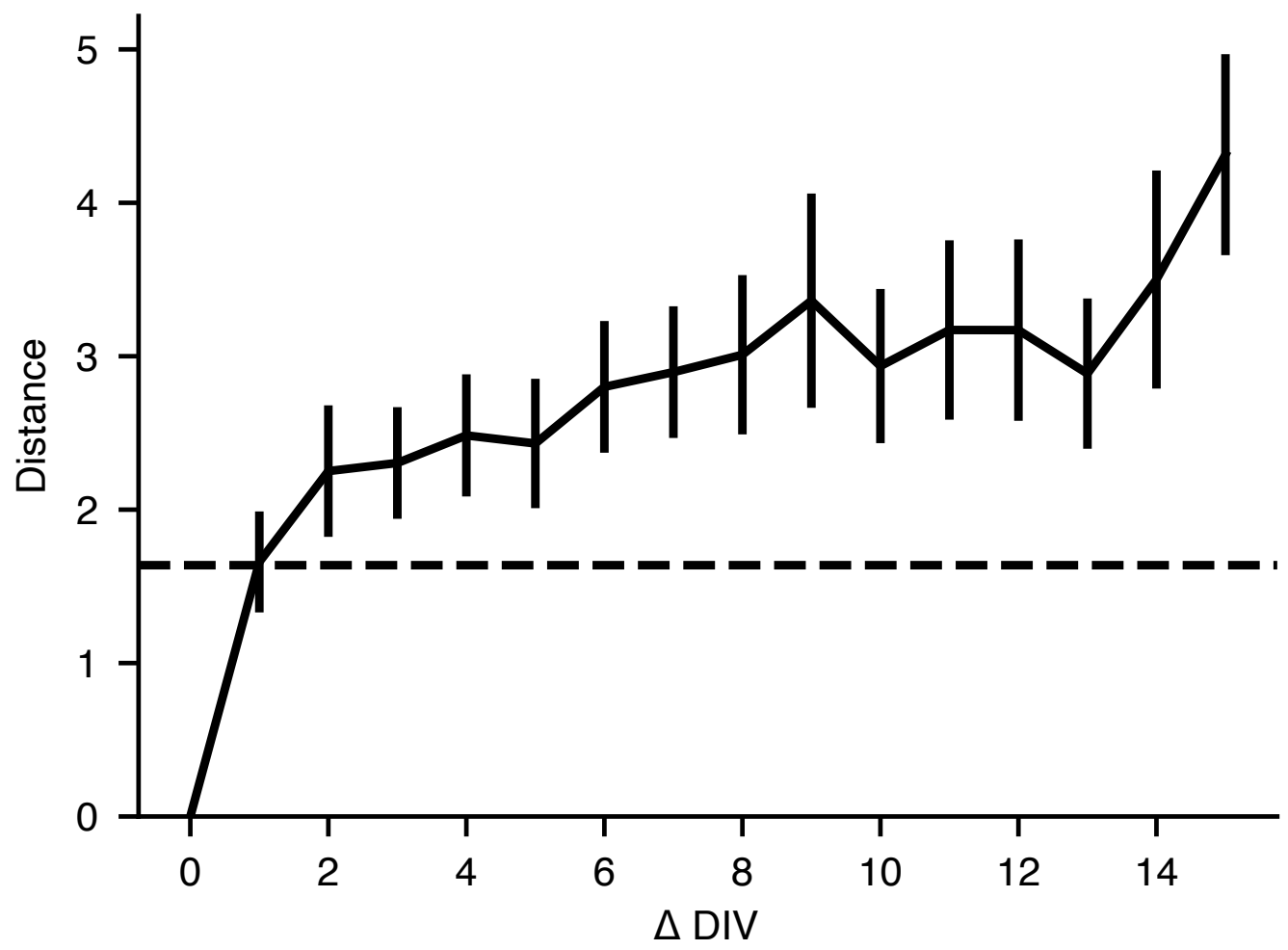

Figure S12: **Burst shape distance grows with the time between recordings of the same culture (Wagenaar dataset).**
Mean Wasserstein distance between burst shapes of recordings from the same culture, as a function of the time gap between recordings. The solid line indicates mean $\pm$ standard error of the mean (N is the number of unique cultures with that time gap; the curve is truncated where $N < 10$). The dashed line indicates the day-over-day distance (smallest possible gap).

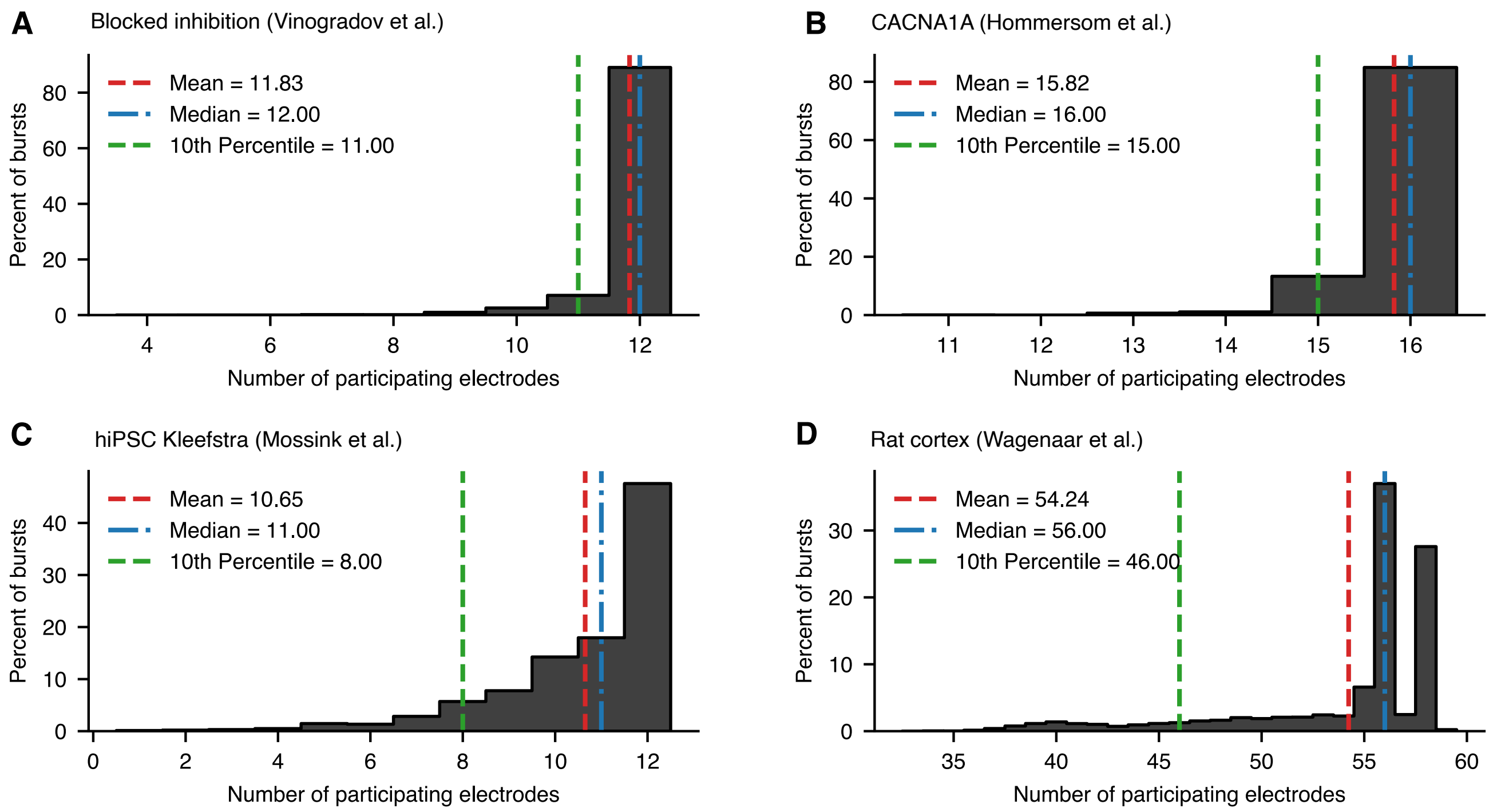

Figure S13: **Across all datasets, in most bursts, the majority of electrodes participate in network bursts, defined as spiking at least once.**
On each panel, the largest number on the x-axis is the total number of electrodes in the corresponding dataset. **(A)** Blocked inhibition: mean=11.83 (98.6%) **(B)** *CACNA1A*$^{+/-}$: mean=15.82 (98.9%) **(C)** hiPSC Kleefstra: mean=10.65 (88.8%) **(D)** Developing rat cortex: mean=54.24 (91.9%).

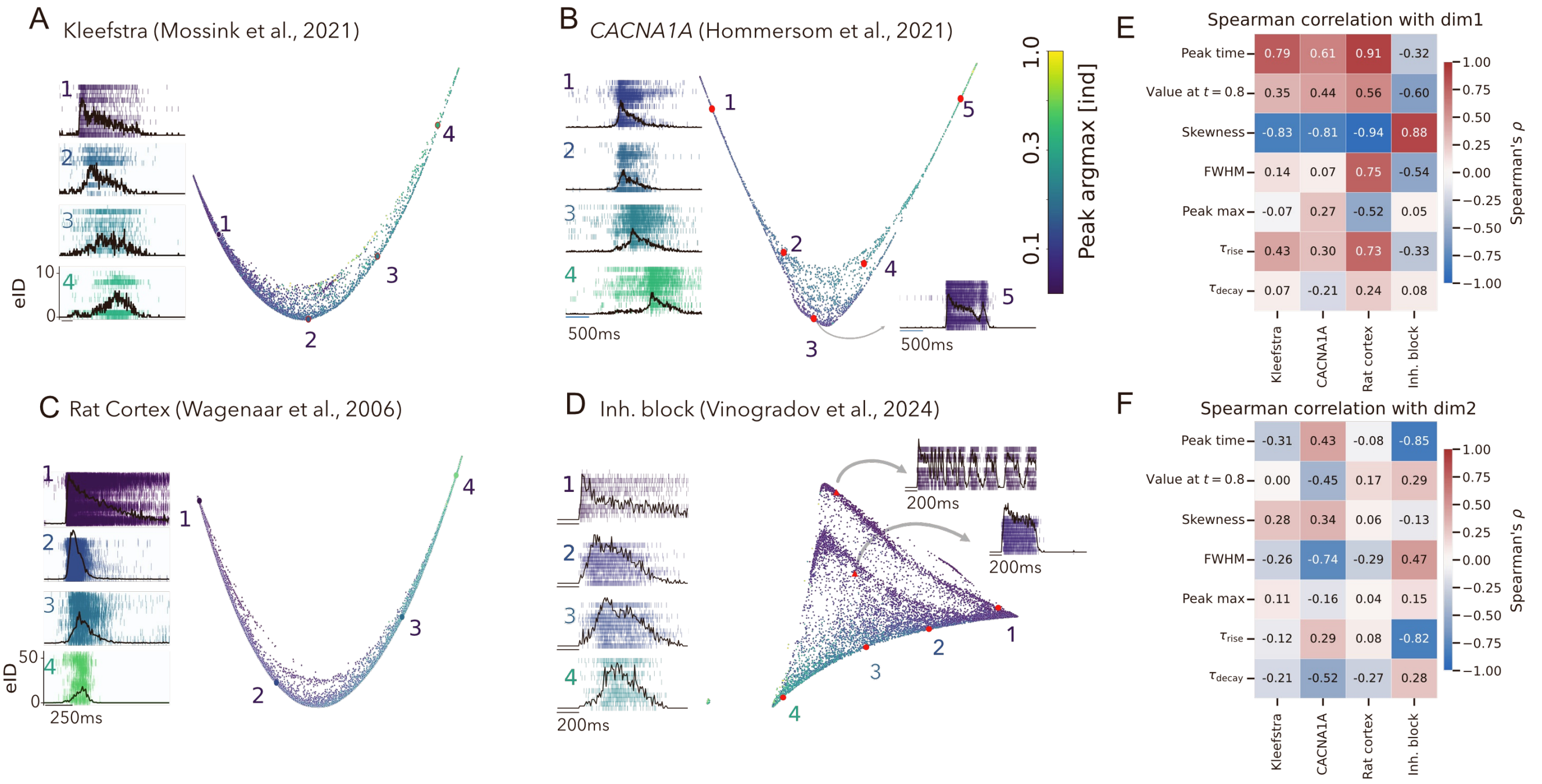

Figure S14: **Burst shape embeddings, examples, and correlations with conventional burst features.**
(**A-D**) Example network bursts and their positions in the first two dimensions of the spectral embedding across datasets. **(E, F)** Correlations of the first two spectral embedding coordinates with conventional burst shape features.
Conventional shape features: The **peak position** was estimated from the smoothed normalized bursts (using a Gaussian filter with $\sigma = 1$). The burst width was quantified by the **full width at half maximum (FWHM)**, **rise and decay time constants**, computed by fitting a parametric rise-decay model, $\hat{y}(t) = b + A\left[1 - \exp\left(-\frac{\max(t-t_0,0)}{\tau_{\text{rise}}}\right)\right]\exp\left(-\frac{\max(t-t_0,0)}{\tau_{\text{decay}}}\right)$, where b is the baseline activity, A is a scale parameter, t_0 is the estimated burst-onset time, and τ_{rise} and τ_{decay} are the rise and decay time constants. The fitted time constants were retained as burst-shape features:$\tau_{\text{rise}}, \tau_{\text{decay}}$. When fewer than three samples preceded the detected peak, only the post-peak part of the waveform was fitted using $\hat{y}(t) = b + A\exp\left(-\frac{t-t_{\text{peak}}}{\tau_{\text{decay}}}\right)$, and τ_{rise} was assigned a missing value. The **skewness** was calculated as $\gamma_w = \frac{\sum_i b_i(t_i-\mu_t)^3}{\left[\sum_i b_i(t_i-\mu_t)^2\right]^{3/2}}$, where b_i is normalized burst, and μ_t is the activity-weighted temporal center $\mu_t = \sum_i b_i t_i$.

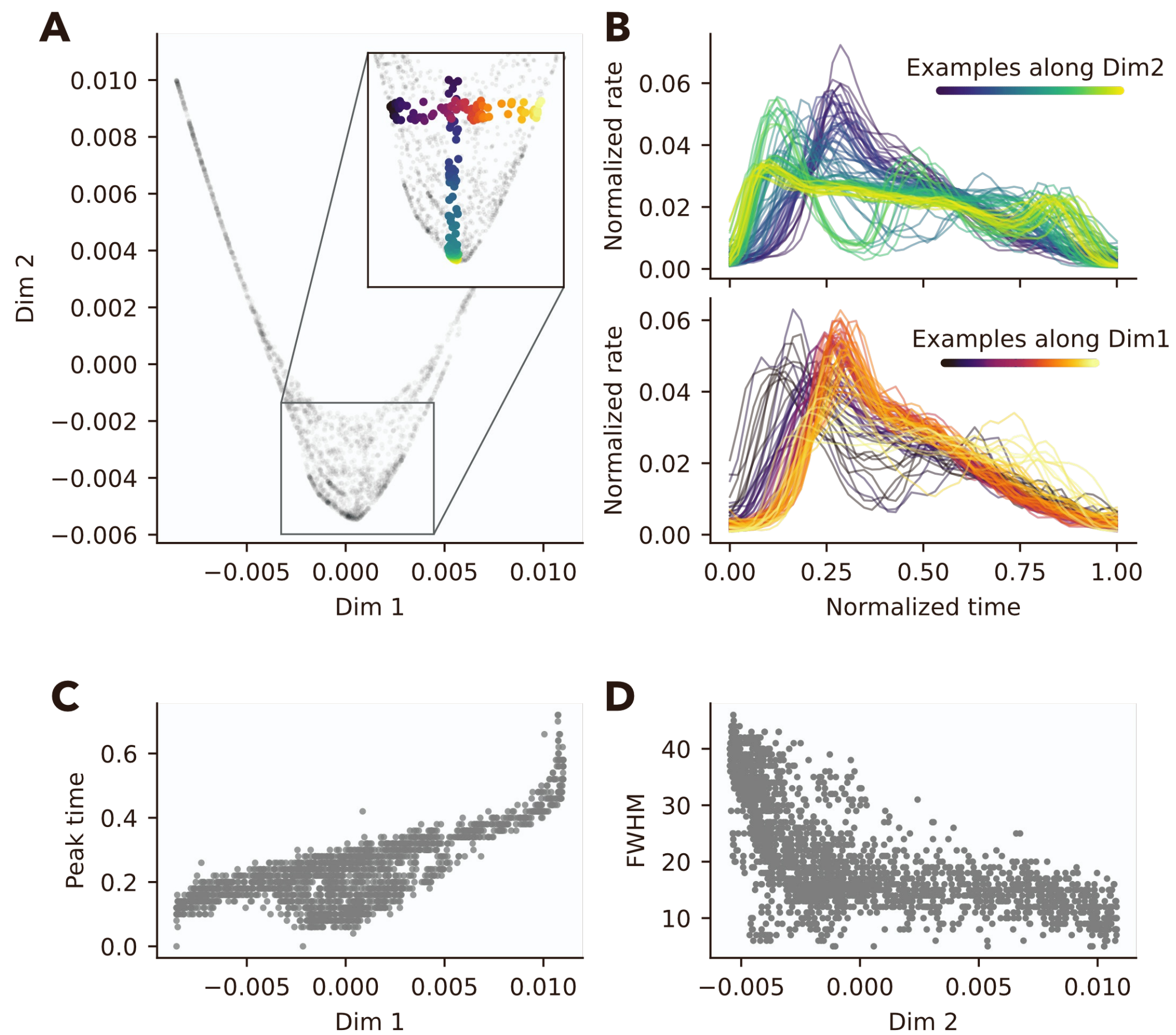

Figure S15: **Burst shapes along the first and second spectral embedding dimensions in the *CACNA1A* dataset.**
(A) Selected points in the spectral embedding. **(B)** Representative burst shapes corresponding to the selected points in panel A. Colors indicate the relative positions of the selected bursts along dimensions 1 and 2. **(C, D)** Peak time and burst width, quantified as the full width at half maximum (FWHM), correlate with the first and second spectral embedding dimensions, respectively.

Supplemental references

[S1] Vinogradov, O., Giannakakis, E., Buendía, V., Uysal, B., Ron, S., Weinreb, E., Schwarz, N., Lerche, H., Moses, E., and Levina, A. (2024). Effective excitability captures network dynamics across development and phenotypes. bioRxiv. doi: 10.1101/2024.08.21.608974.

[S2] Hommersom, M.P., Doorn, N., Puvogel, S., Lewerissa, E.I., Mordelt, A., Ciptasari, U., Kampshoff, F., Dillen, L., Van Beusekom, E., Oudakker, A., Kogo, N., Dolga, A.M., Frega, M., Schubert, D., Van De Warrenburg, B.P.C., Nadif Kasri, N., and Van Bokhoven, H. (2025). *CACNA1A* haploinsufficiency leads to reduced synaptic function and increased intrinsic excitability. Brain *148*, 1286–1301. URL: https://academic.oup.com/brain/article/148/4/1286/7832339. doi: 10.1093/brain/awae330.

[S3] Wagenaar, D.A., Pine, J., and Potter, S.M. (2006). An extremely rich repertoire of bursting patterns during the development of cortical cultures. BMC Neuroscience *7*, 11. doi: 10.1186/1471-2202-7-11.

[S4] Mossink, B., Verboven, A.H., Van Hugte, E.J., Klein Gunnewiek, T.M., Parodi, G., Linda, K., Schoenmaker, C., Kleefstra, T., Kozicz, T., Van Bokhoven, H., Schubert, D., Nadif Kasri, N., and Frega, M. (2021). Human neuronal networks on micro-electrode arrays are a highly robust tool to study disease-specific genotype-phenotype correlations in vitro. Stem Cell Reports *16*, 2182–2196. URL: https://linkinghub.elsevier.com/retrieve/pii/S221367112100326X. doi: 10.1016/j.stemcr.2021.07.001.
